# Toward the discovery of biological functions associated with the mechanosensor Mtl1p of *Saccharomyces cerevisiae* via integrative multi-OMICs analysis

**DOI:** 10.1038/s41598-021-86671-8

**Published:** 2021-04-01

**Authors:** Nelson Martínez-Matías, Nataliya Chorna, Sahily González-Crespo, Lilliam Villanueva, Ingrid Montes-Rodríguez, Loyda M. Melendez-Aponte, Abiel Roche-Lima, Kelvin Carrasquillo-Carrión, Ednalise Santiago-Cartagena, Brian C. Rymond, Mohan Babu, Igor Stagljar, José R. Rodríguez-Medina

**Affiliations:** 1 Department of Biochemistry, Medical Sciences Campus, University of Puerto Rico, San Juan, PR 00936-5067 USA; 2 Comprehensive Cancer Center, University of Puerto Rico, Puerto Rico Medical Center, Rio Piedras, PR 00936-3027 USA; 3 Department of Biology, University of Kentucky, Lexington, KY 40506 USA; 4 Department of Biochemistry, University of Regina, Regina, SK S4S 0A2 Canada; 5 Donnelly Centre, Department of Biochemistry, Department of Molecular Genetics, University of Toronto, Toronto, ON M5S 3E1 Canada; 6 Mediterranean Institute for Life Sciences, Split, Croatia

**Keywords:** Biochemistry, Biological techniques, Cell biology, Computational biology and bioinformatics, Microbiology, Molecular biology, Systems biology

## Abstract

Functional analysis of the Mtl1 protein in *Saccharomyces cerevisiae* has revealed that this transmembrane sensor endows yeast cells with resistance to oxidative stress through a signaling mechanism called the cell wall integrity pathway (CWI). We observed upregulation of multiple heat shock proteins (HSPs), proteins associated with the formation of stress granules, and the phosphatase subunit of trehalose 6-phosphate synthase which suggests that *mtl1Δ* strains undergo intrinsic activation of a non-lethal heat stress response. Furthermore, quantitative global proteomic analysis conducted on TMT-labeled proteins combined with metabolome analysis revealed that *mtl1Δ* strains exhibit decreased levels of metabolites of carboxylic acid metabolism, decreased expression of anabolic enzymes and increased expression of catabolic enzymes involved in the metabolism of amino acids, with enhanced expression of mitochondrial respirasome proteins. These observations support the idea that Mtl1 protein controls the suppression of a non-lethal heat stress response under normal conditions while it plays an important role in metabolic regulatory mechanisms linked to TORC1 signaling that are required to maintain cellular homeostasis and optimal mitochondrial function.

## Introduction

The fungal cell wall is a physical structure that evolved to shield cells from the changing conditions of the ecological niches in which they thrive*,* acting as a porous barrier that isolates the delicate plasma membrane (PM) and cytoplasm from the external environment^1,2^. Fungi have developed a molecular response mechanism named the Cell Wall Integrity Pathway (CWI) to maintain cellular integrity in response to changes in the cellular environment^3–9^. In the CWI, information conveying environmental changes such as chemicals, biomolecules, ions, radiation, osmotic pressure, or thermal motion enters the cell by way of stress mechanosensor proteins embedded within the cell wall-PM composite^4^.

In the budding yeast *Saccharomyces cerevisiae*, the CWI pathway participates in regulation of cellular processes including cell wall biosynthesis, cell wall repair, and maintenance of cell integrity^1,2,4,8,10,11^, response to oxidative stress^12,13^, heat shock^5,14–16^, hypo osmotic shock^6^, glucose starvation and regulation of key cellular metabolic processes such as ribosome assembly and energy metabolism in response to nutrient starvation^7,17,18^, impaired cell wall synthesis^8^, antifungal drug treatments, and other environmental stresses that can alter the integrity of the cell wall^12,19^.

The CWI communicates stress signals via five transmembrane mechanosensory proteins consisting of the Wsc-family (Wsc1p, Wsc2p and Wsc3p), and Mid-family (Mid2p and its homologue Mtl1p)^4,8,20,21^. Wsc1p has been associated with activation of the CWI pathway in response to Caspofungin^22^, alkaline pH^23^ and reorganization of actin during hypo osmotic stress^24^ while Mid2p has been associated with activation in response to Calcofluor white^8,25^, mating pheromone^26^, vanadate^27^, and acidic conditions^28^, and oxidative stress^13^. The *WSC2* and *WSC3* genes act as multi-copy suppressors in mutants with glycerol synthesis abnormalities^29,30^ and Mtl1p has been associated with the response to hydrogen peroxide-induced oxidative stress and glucose starvation^12,31^.

These proteins communicate physical and chemical signals to the cell interior via their cytoplasmic domains, by activating a set of downstream effectors that act in a cascade–like fashion^12,32^. As an initial step, Rom2p is a Rho1p GEF that attaches to the cytoplasmic tail of these mechanosensor proteins in a regulated manner to activate Rho1p GTPase activity which in turn activates Pkc1p^33^. The ensuing sequential signaling cascade includes kinases Bck1p, Mkk1p, Mkk2p and the end-kinase Slt2p. Once a mechanosensor activates the CWI pathway, and Slt2p is hyperphosphorylated, it translocates to the nucleus and activates transcription factor Rlm1p and the heterodimer complex Swi4p/Swi6p by phosphorylation^34^. These transcription factors induce the transcription of specific genes for the synthesis of cell wall components, osmo-protective molecules, and cell cycle regulatory proteins^34–36^. According to Harrison and co–workers, a linear signal transmission scheme like this cannot be used exclusively to explain every instance of CWI pathway activation^37^. They proposed that in some cases, lateral signals could bypass elements upstream of Pkc1p through non-linear mechanisms that activate the cell integrity MAPK pathway such as: Pkc1p by actin stress, Bck1p by osmotic stress, and Mkk1p, Mkk2p and Slt2p by heat stress^37^. Others have proposed that Rom2p may also mediate stress responses with the involvement of cAMP and Ras2p^32^. This observation was subsequently integrated in a model where Mtl1p is proposed to activate a general stress response to glucose starvation and oxidative stress through Tor1p and Ras2p inhibition^31^. We have recently provided direct evidence that Ras2p can physically interact with Wsc1p, Mid2^13^and Wsc2^38^ which supports the model described by Petkova that Ras2p is involved in regulation of Mtl1p stress signaling.

Although the functional roles of Wsc- and Mid-family mechanosensors may appear to be redundant^4,19^, Mtl1p has been designated with unique roles responsible for the response to H_2_O_2_-induced stress^12^, also reported for its homolog Mid2p^13^, activation of the CWI pathway in response to glucose starvation^31^, extension in chronological life span, and the integrity of mitochondrial function in yeast cells^39^. The mechanism by which Mtl1p exerts its protective roles is not fully understood although in a study by Petkova et al., they report a “genome-wide transcriptional analysis revealed a cluster of protective stress genes that were down-regulated in the absence of Mtl1p”^31^. These investigators used microarray analysis to quantify mRNA expression (Affymetrix GeneChip Yeast Genome S98 array) contrasting with more comprehensive analytical methods used in this study. To expand these previous studies, we have therefore designed a strategy for identification of components of the biological network of Mtl1p by analyzing the macromolecular changes occurring at the transcriptome and proteome levels when the *MTL1* gene is deleted with further analysis of the systematic effects of these macromolecular changes through metabolite analysis to pinpoint the focus of the mutation.

In this manuscript, we present the findings of our studies on the mechanosensor Mtl1p of *S. cerevisiae* using a multipronged Omics approach. We have conducted quantitative analyses that elucidated the transcriptome, proteome, and metabolome profiles of *mtl1Δ* strains compared to wild-type controls under normal culture conditions to uncover the biological networks requiring Mtl1p for maintaining metabolic homeostasis and resistance to stress conditions. Because stress–response mechanisms are well conserved, the knowledge derived from experiments using *Saccharomyces cerevisiae* as a model system provides insights into the mechanisms by which opportunistic fungal pathogens adapt to environmental stresses and can have important translational applications for antifungal drug development^40,41^.

## Materials and methods

### Strains

All *Saccharomyces cerevisiae* strains used in this study.

#### Confirmation of *MTL1* deletion strain

The BY4742 wild-type (WT) and *YGR023w (mtl1Δ)* strains were obtained from Open Biosystems. The chromosomal deletion of *MTL1* was confirmed by PCR with genomic DNA. These strains were used in transcriptome, proteome, and metabolome experiments.

#### Cell culture conditions

The *S. cerevisiae* WT and *mtl1Δ* strains were cultured overnight at 27 °C, with shaking at 210 rpm, in 25 mL of complete synthetic medium (CSM) containing all amino acids and 2% glucose (Sigma), plus 0.67% Nitrogen base supplemented with ammonium sulfate (Fisher Scientific). Two to three cultures prepared with different media batches were used per experiment. The next day, the cultures were replenished with fresh CSM and cultured at 27 °C until they reached an Optical Density (OD_600_) between 0.6 and 0.7 units. The cells were harvested by centrifugation at 3838xg for 5 min at 4 °C, cell pellets were washed with 1 ml ice-cold sterile deionized water, and centrifuged as before. Cell pellets were suspended in the appropriate buffer solutions described below for RNAseq, Total Protein, and Total Metabolite extractions, or stored for future use at – 80 °C.

### RNAseq analysis

#### Transcriptome library construction and Ion Proton sequencing

Cells pellets were resuspended in 2 ml freshly prepared Buffer Y1 (1 M sorbitol, 0.1 M EDTA, pH 7.4, and 0.1% ß-Mercaptoethanol (Sigma M-3148, 98%) with 50 U Zymolase added per 1 × 107 cells (ICN Code 320921 Zymolase Arthrobacter luteus 20,000U/g) and incubated for 60 min at 30 °C with gentle shaking to generate spheroplasts. These were centrifuged for 5 min at 3000×*g* at room temperature (RT) and the supernatant was discarded. Spheroplast pellets were used for mRNA isolation.

Total RNA was isolated from *S. cerevisiae* WT and *mtl1Δ* strains using the Qiagen RNeasy kit. RNA quality was verified using the Agilent RNA 6000 Nano Kit in the 2100 Bioanalyzer from Agilent Technologies following the RNA 6000 Nano Kit user manual. This was followed by the isolation of mRNA from 5.0 µg of total RNA using the NEBNext Poly (A) mRNA Magnetic Isolation Module (NEB Cat. No. E7490S, Version 6.0). The mRNA quality was assessed using the Agilent RNA 6000 Pico Kit in the 2100 Bioanalyzer from Agilent Technologies following the Agilent RNA 6000 Pico Kit user manual.

Transcriptome libraries were prepared using the Ion Total RNA-Seq Kit v2 from Life Technologies (Publication Number 4476286). The mRNA samples were fragmented by RNase III digestion for 3 min at 37 °C and purified according to Ion Total RNA-Seq Kit v2 manual specifications. Sample yield and size distribution were determined using the RNA 6000 Pico Kit with the Agilent 2100 Bioanalyzer. Three mRNA samples each from wild-type and *mtl1Δ* strains (6 different biological samples in total) were analyzed by NGS (Next Generation Sequencing). Each of the six samples was run individually in a chip (hence they were not barcoded). Additionally, one chip was run with all the samples mixed. For this experiment samples were barcoded using Ion Xpress RNA-Seq Barcode 1–16 Kit from Life Technologies. Emulsion PCR and enrichment steps were carried out in the Ion OneTouch 2 System and in the Ion OneTouch ES System, respectively, using the Ion PGM Hi-Q View OT2 Kit from Life Technologies following the Ion PGM Hi-Q View OT2 Kit User Guide (Publication Number MAN0014580). After the emulsion PCR, an aliquot was analyzed in the Qubit 2.0 Fluorometer (Invitrogen) using the Ion Sphere Quality control kit from Life Technologies. The empty ISP’s were removed during the enrichment process which employs magnetic Dynabeads MyOne Streptavidin C1 Beads (Invitrogen) to immobilized Templated ISP’s.

Each sample was run in the Ion PGM Sequencer using the Ion 318 Chip V2 BC from Life Technologies. The samples were prepared using the Ion PGM Hi-Q View Sequencing Kit following the Ion PGM Hi-Q View Sequencing User Manual (Publication Number MAN0014583). Seven chips were loaded in total and run in the Ion PGM Sequencer.

The RNAseq reads were processed and analyzed with the CLC genomics workbench v12 [www.qiagenbioinformatics.com] to obtain the expressed transcriptomes/genes. Firstly, the raw reads were processed from the original *fastq* files using the Quality Control analysis pipeline, which is used to determine the quality of the reads and correctly trim the sequences. Then the CLC read alignment analysis pipeline was performed using the *S. cerevisiae* S288c as the reference genome. The *Total Counts* result per gene was used as the gene/transcriptome expression value. This process was repeat to obtain the six results that corresponds to wild type group (3 replicates) and mutant type group (3 replicates).

#### Bioinformatics analysis of differently expressed mRNAs

To determine the differently expressed genes/transcriptomes, the mutant and wild type replicated groups were considered as experimental and control, respectively. Python^42^ and R [www.R-project.org] tailored pipelines were implemented for dataset pre-processing. These pipelines were used to detect missing values and identify outliers. Then the Interquantile (IQR) mean method was used for outlier imputations. Based on the dataset and expected results, a single channel model design comparison was used, which involves two samples, *Experimental Group vs Control Group.* R Bioconductor software *limma* version 3.9.0 (https://www.bioconductor.org)^43^ was applied to implement and run the model. Limma then used an empirical Bayes function that performed the statistical analysis of interest. The output of this procedure was a data frame that especially contained for each protein its fold-change (FC), and ordinary moderated p-values, in our case Benjamini–Hochberg adjustment.

### Quantitative proteomics by tandem mass tag (TMT) labeling

#### Sample processing

Five each of WT control and *mtl1Δ* experimental total protein samples (250 μg per sample) were delivered for quantitative proteomics analysis (Supplementary Table S3). Total protein lysates for WT and *mtl1Δ* strains were generated from cells pellets disrupted with glass beads in lysis buffer (50mMTris HCl pH7.5, 10% Glycerol, 1% Triton X-100, 0.1% SDS, 150 mM NaCl, 5 mM EDTA, 5X Protease Inhibitor Cocktail and 5X PMSF) by vortexing for 60 s at high speed alternating with 3 min on ice, repeated for 4 cycles. Total protein concentration was quantified by the DC-Protein Assay (Bio-Rad).

Sample processing began with an acetone precipitation overnight with 100 μg of total protein to concentrate the samples on the next day and wash away unwanted substances. Samples were resuspended in 50 μl of 2X sample buffer (95% Laemli/ 5% β-mercaptoethanol). SDS-PAGE using Mini-PROTEAN TGX Precast Gel (12% fixed gel) was allowed to run for 15–20 min at 150 V to generate a 1.5 cm band. Gels were stained with Bio-Safe Coomassie G-250 to visualize the quality of the proteome bands present in each lane, and to be able to cut them out. After the proteome gel bands were cut out, they were destained using a 50 mM ammonium/ 50% Acetonitrile solution at 37 °C. Then, they were reduced with Dithiothreitol (25 mM DTT in 50 mM Ammonium Bicarbonate) at 55 °C, alkylated with Iodoacetamide (10 mM IAA in 50 mM Ammonium Bicarbonate) at room temperature in the dark, and digested with trypsin (Promega) overnight at 37 °C. Digested peptides were extracted out of the gel pieces using a mixture of 50% acetonitrile/ 2.5% formic acid in water. Extracted samples were dried and stored at − 80 °C to wait for TMT labeling procedure and subsequent LC–MS/MS analysis.

#### TMT labeling and fractionation

As specified by the manufacturer’s protocol, dried extracted samples were reconstituted in 100 mM TEAB and labeled with the TMT 10plex labeling reagents (41 μl, 0.8 mg). The TMT labels were added as described in Table 1. The addition was followed by an hour incubation allowing the labelling reaction to occur, and a quenching step of 15 min. Finally, equal amounts of each sample were mixed to generate a final pool. We used 75% of the volume per sample for this pool, and kept the remaining 25% of the sample volume in each individual vial which is now stored in our freezer in case the analysis has to be repeated. The final pool was dried to proceed with the fractionation procedure.

**Table 1:** *Saccharomyces cerevisiae* strains.

Strain	Genotype	Source
BY4742	*MAT α his3Δ1 leu2Δ0 lys2Δ0 ura3Δ0*	Open Biosystems
YGR023w	*MAT α his3Δ1 leu2Δ0 lys2Δ0 ura3Δ0 mtl1::KanMx4*	Open Biosystems

Fractionation was performed using the “Pierce High pH Reversed-Phase Peptide Fractionation Kit” and following the manufacturer’s instructions. Briefly, the column was conditioned twice using 300 μl of Acetonitrile, centrifuging at 5000×*g* for 2 min; and repeating the steps using 0.1% Trifluoroacetic acid (TFA). Sample (final pool) was bound to the column, washed to remove contaminants and any unbound TMT reagent, and eluted 8 times into 8 different vials using a series of elution solutions with different Acetonitrile/TFA percentages. Elution solutions are specified in the manufacturer’s protocol. The entire procedure was performed twice and 16 fractions were generated followed by drying and LC–MS/MS analysis.

#### Sample preparation for LC MS/MS

The reconstitution of the fractions for mass spectrometry analysis was made using 2.5% acetonitrile/2.5% formic acid in water. A total of 3 μl were transferred to a special sample vial to be able to inject 2 μl of sample into the instrument. The remaining volume of the reconstituted fractions were stored at – 80° C.

#### LC–MS/MS analysis

For peptide separation on an Easy-nLC1200 instrument (Thermo Fisher Scientific), a PicoChip H354 REPROSIL-Pur C18-AQ 3 μm 120 A (75 μm × 105 mm) chromatographic column (New Objective) was used. The separation was obtained using a gradient of 7–25% of 0.1% of formic acid in acetonitrile (Buffer B) for 102 min, 25–60% of Buffer B for 20 min, and 60–95% Buffer B for 6 min. Making a total gradient time of 128 min at a flow rate of 300 nl/min, with an injection volume of 1 μL per sample.

Q-Exactive Plus (Thermo Fisher Scientific) operates in positive polarity mode and data-dependent mode. The full scan (MS1) was measured over the range of 375 to 1400. The MS2 (MS/MS) analysis was configured to select the top ten most intense ions for HCD fragmentation, configured over the range of 200 to 2000 m/z. A dynamic exclusion parameter was set for 30.0 s.

#### Database search

Once the Mass Spectrometry analyses were finished, the raw data files were searched with a *S. cerevisiae* database and downloaded from UniProt (Universal Protein Resource). The raw data was analyzed with Proteome Discoverer software version 2.1 (https://www.thermofisher.com/pr/en/home/industrial/mass-spectrometry/liquid-chromatography-mass-spectrometry-lc-ms/lc-ms-software/multi-omics-data-analysis/proteome-discoverer-software.html) using workflows configured for quantitative proteomics. A dynamic modification for oxidation + 15.995 Da (M) was configured. A static modification of + 57.021 Da (C) generated by the alkylation during processing, and static modifications from the TMT reagents + 229.163 Da (Any N Term, K) were all included in the parameters for the search.

#### Quantitative proteomics by TMT labeling

The analysis was performed for the datasets related to Mutant (*mtl1Δ*) and Wild Type (WT) groups to determine the differential protein abundances. The comparisons included five replicates for both *mtl1Δ* and WT groups as described in Supplementary Table S3, where the Control Group and Experimental Group correspond to MT and WT replicated samples, respectively. Similar to our analysis of differently expressed mRNAs, the datasets were pre-processed with tailored Python^42^ and R [www.R-project.org] scriptings for missing values, as well as identification and processing of outliers using the Interquantile (IQR) mean imputation method. A single channel design for the Experimental Group vs Control Group was implemented and ran using the R Bioconductor software *limma* version 3.9.0 (https://www.bioconductor.org)^43^. The results from the statistical analysis of significantly different protein abundances were considered based on a Fold Change FC ≥ 2 and adjusted *P* value (using the Benjamini–Hochberg adjustment) ≤ 0.05.

### Metabolomics analysis

#### Sample Processing

Cells pellets of WT and *mtl1Δ* strains were resuspended in 1 ml of pure methanol (Fisher HPLC grade) pre-chilled at − 20 °C, for 5 min (quenching step). The cell suspensions were centrifuged for 5 min at 167×*g*, 4 °C. The methanol supernatant was removed from each sample with a Pasteur pipet. The samples were resuspended in 1 ml of a 1:1 mixture of methanol and sterile deionized water and homogenized with a Poly Tron PT-2100 homogenizer (set at 15) using two 7-s pulses.

#### Derivatization of glucose

Metabolic extracts from the WT and *mtl1Δ* strains (n = 13, each) were collected and dried in a nitrogen gas stream at 50 °C (RapidVap, Labconco). Nitrilation was performed as previously described^44^ by adding 150 μl of 0.2 mM hydroxylammonium chloride in pyridine to the dried sample, and then heating at 90 °C for 40 min. After that, acetylation was performed by adding 250 μl of acetic anhydride, and then heating at 90 °C for 60 min. After that, the sample was dried in a nitrogen gas stream again, and then redissolved in 400 μl of ethyl acetate. Derivatized samples were collected and stored at − 20 °C.

#### Derivatization of other metabolic features

Metabolic extracts from the WT and *mtl1*Δ strains (n = 7, each) were collected, evaporated to dryness in a nitrogen stream, derivatized by methoxyamination by adding 50 μl of 20 mg/ml solution of methoxyamine hydrochloride in pyridine (Sigma-Aldrich) and incubated at 37 °C for 2 h. Trimethylsilylation was subsequently performed by adding 50 µl of N-methyl-N-trimethylsilyl-trifluoroacetamide (MSTFA + 1% TMCS, Sigma-Aldrich) and incubated for 1 h at 65 °C. Samples were centrifuged at 15.700 × g for 10 min at RT. Supernatants were transferred to glass vials and stored at − 20 °C.

#### Analysis of glucose content

Twenty microliters per sample were added to glass vials with inserts to evaluate the glucose content via GC–MS (GC/MS-TQ8050, Shimadzu Inc.) using SIM mode and analytical conditions previously described^44^. The glucose concentration in the samples was performed by comparing the obtained absorbance values in each sample with the glucose (Sigma) calibration curve.

#### Analysis of other metabolites

Twenty microliters per sample were added to glass vials with inserts followed by the addition of 1 mM 2-fluobiphenyl (Sigma-Aldrich) as an internal standard. Samples were processed via GC/MS-TQ8050 using full scan mode and analytical conditions as previously described^45^. Peak integration was performed using GCMS Labsolution data analysis version 4.45 software (https://www.ssi.shimadzu.com/products/gas-chromatography-mass-spectrometry/gcmssolution-software.html). Mass spectral library searches of the major chromatographic peaks were conducted using the GCMS Lab solution data analysis software equipped with the NIST14/2014/EPA/NIH database in each data set, which resulted in a final data set consisting of 45 metabolic features selected for the metabolomics analysis. Quantitative analysis of metabolic features in each sample was performed by calculation of a response factor using the internal standard 1 mM 2-fluobiphenyl spiked into each sample before the GC/MS analysis.

#### Quality assessment and quality controls in metabolomics analysis

Reproducibility of metabolite recovery, the performance of sample extraction, derivatization, and instrumentation were validated by the utilization of several blank samples, including a system suitability blanks, and derivatization processing blanks. To evaluate analytical accuracy and precision, an external quality assessment was performed using 1 mM 2-fluobiphenyl spiked into derivatization blank samples before running on the GC/MS (n = 3). The percent of relative standard deviation (%RSD) of 2-fluobiphenyl peak abundances accounted for 3.8%, which demonstrates good reproducibility of the method. For systematic bias mitigation, we performed the randomization of the sample analysis order. Blanks and quality control samples were spaced evenly among the injections to monitor instrument stability.

#### Bioinformatic analysis of metabolites

Metaboanalyst 4.0^46,47^ was used for bioinformatic analysis. Identified concentrations of each metabolite were composed as the data matrix and processed. Data integrity check was performed according to default settings on the Metaboanalyst interface and normalized by OD, and range scaled to improve the pattern recognition for metabolomics data. Differences between WT and *mtl1*Δ strains were evaluated using Orthogonal Projections to Latent Structures Discriminant Analysis (OPLS-DA). Quality and reliability were assessed by cross-validation by using two parameters: the R2(Y) (a measure of how much variation is represented by the model) and Q2(X) (a measure of how accurate the data are classified in the model)^48^. To evaluate the model performance, class labels were permuted 1000 times to identify whether differences between groups were significant. Mann–Whitney *U* test was used to identify statistically significant (p < 0.05) metabolic features.

#### Functional enrichment analysis of proteome and metabolome data

To understand the cellular regulatory mechanisms in *mtl1*Δ strain, we applied an integrative multi-omics analysis of *mtl1*Δ proteome, and metabolome using Cytoscape and the ClueGO plugin in the Cytoscape which integrates Gene Ontology (GO) terms and creates a functionally organized GO/pathway term network corrected by kappa statistics^49^ using two-sided (Enrichment/Depletion) tests based on the hypergeometric distribution (Supplementary Table S2). The *P* value < 0.05 was corrected by Bonferroni step down correction method; Min GO Level = 7 and Max GO Level = 16; Kappa Score Threshold = 0.4; Initial Group Size = 2 and sharing Group Percentage = 50.0. The created network represented the pathways as randomly colored nodes, where some of them were partially overlapped depending on their functional relation. The node size represented the term enrichment significance and is highlighted by a large name label. Edges indicate statistically significant associations between GO terms. Since ClueGo mostly evaluates functional enrichment, taking into account the number of analyzed genes/proteins, therefore, to evaluate other functional enrichments where a small number of proteins per term existed, we applied the GeneMANIA plugin in the Cytoscape to model possible interaction networks. GeneMANIA integrates GO terms and creates a functionally organized GO/pathway term network applying Q-values from an FDR corrected hypergeometric test for enrichments and the Benjamini–Hochberg corrections^50^. Pathways visualizations have been carried out using ClueGO plugin for Cytoscape 3.7.2 and ChemDraw 15 (PerkinElmer).

## Results and discussions

### Integrative analysis of transcriptome and proteome data

**Figure 1:**
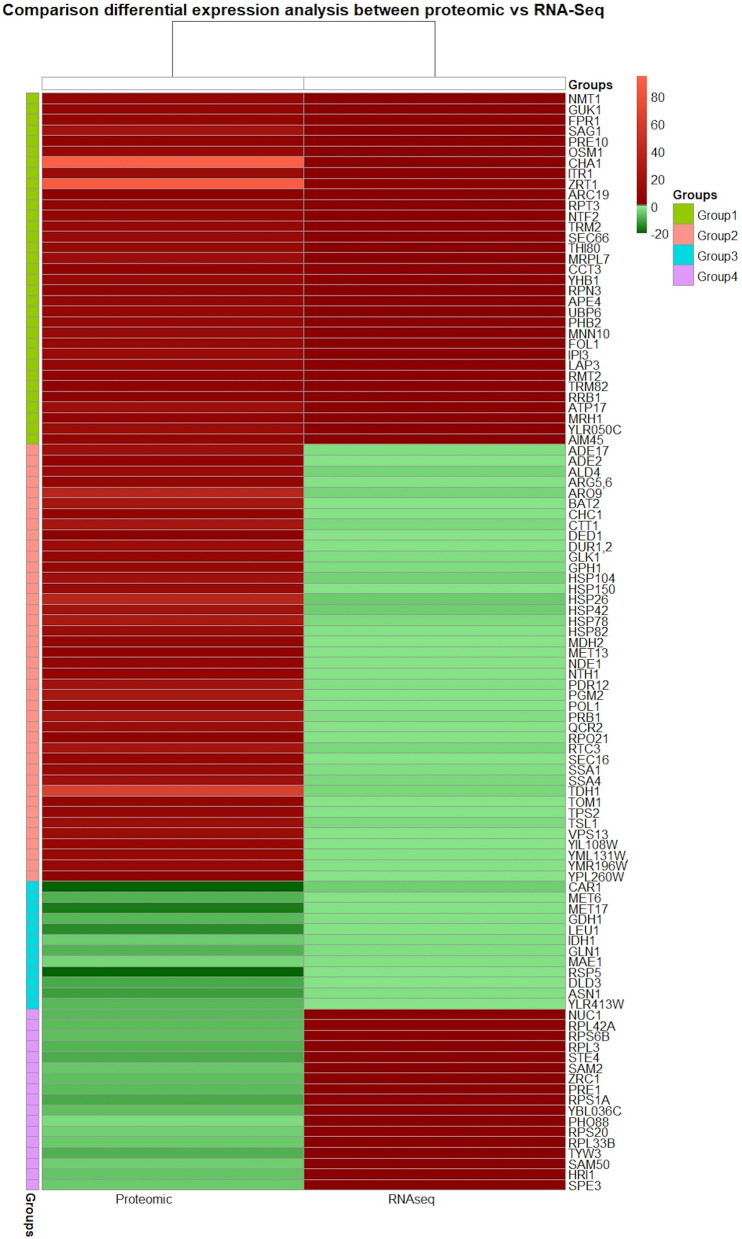
Comparison of differential protein and mRNA expression profiles. (Left panel, Proteomic_FC) 104 proteins identified on the right margin, with statistically significant changes in abundance of twofold or greater in the *mtl1Δ* strain are shown alongside their mRNAs (Right panel, RNAseq_FC) with expression changes of twofold or greater. Note that a significant block of proteins correlated with their mRNA levels, while others did not correlate (see Results and Discussion for details). The color scale indicates relative fold-change (Red = FC $\ge$ 2, Green = or FC $\le$ − 2), $P \: \mbox{value} \le$ 0.05.

To assess the effect of Mtl1p in the regulation of stress genes, we measured the mRNA levels in the *mtl1Δ* strain compared to a WT using Next Generation Sequencing (NGS) and quantitative proteomic analysis by TMT labelling of tryptic peptides generated from total proteins followed by tandem mass spectrometry (MS–MS). After we filtered the results of these experiments according to the selected criteria (FC ≥ 2 or FC ≤ − 2 $P \: \mbox{value} \le$ 0.05) we identified a total of 104 differentially regulated mRNA transcripts and proteins (Supplementary Table S1, Fig. 1)*.* Interestingly, 22 down-regulated ORFs reported in this study were also reported in a previous study^31^. We found 33 upregulated (Group 1) and 12 downregulated proteins (Group 3) that correlated at both the mRNA and protein levels, while there were 42 upregulated (Group 2) and 17 downregulated proteins (Group 4) that were uncorrelated with their corresponding mRNAs (Supplementary Table S1). One protein that was differentially regulated in the *mtl1Δ* strain (Group 2) was the catalytic subunit of the β-1,3-glucan synthase, Fks1p, a protein induced by the CWI pathway in response to cell wall damage (Supplementary Fig. S1). In the *mtl1Δ* strain, this gene exhibits repressed mRNA levels in the RNAseq data yet proteome analysis shows a sevenfold increase in expression this protein (mRNA -2.52 vs Protein + 7.07). Remarkably, previous breakthrough studies using transcriptome and proteome data from *E. coli* and yeast reported that protein and mRNA copy numbers for any given gene may be uncorrelated, highlighting the disconnect between proteome and transcriptome quantitative analysis in single cells^51,52^, which is also likely to apply to global quantitative analyses used here. Contributing factors to the uncorrelated behavior include an undervalued role for post-transcriptional, translational, and protein turnover regulation in the determination of protein concentrations^53^.

Although we did not find a mutual relationship in expression between the mRNA and proteins in all cases, genes associated with the stress response such as *SSA1, SSA4, HSP26, HSP42, HSP78, HSP82, HSP104,* and *CTT1* were upregulated at the protein level. Another finding was that the proteins encoded by genes *MRPL7, PHB2, ATP17, AIM45, ALD4, HSP78, HSP82, NDE1*, and *QCR2*, all of which encode components of the mitochondria, were upregulated at the protein level in the *mtl1Δ*, while mitochondrial components *IDH1, MAE1, NUC1*, and *SAM50* were downregulated (Fig. 1, Supplementary Table S1). A review that addressed the question regarding the inverted correlation between transcripts and proteins suggests that despite the effectiveness of mRNA expression values shown in a variety of applications, their correlations with protein levels are almost certainly only correlative rather than causative^53^. Therefore, it is most probable that the concentration of proteins and their interactions represent true contributing forces in the cell^54^.

The cAMP/PKA pathway regulates genes containing a Stress Response Element (STRE) consisting of the sequence 5′-CCCCT-3′ bound by transcription factors (TFs) Msn2p and Msn4p^17^. Activation of PKA by increasing intracellular cAMP levels inhibits the accumulation of carbohydrate stores, respiratory growth, and the transcription of genes bearing an STRE through negative regulation of the Rim15p kinase by PKA and inhibition of nucleocytoplasmic shuttling by TFs Msn2p and Msn4p^55,56^. To test if the regulated mRNAs in the *mtl1Δ* strain represent genes with STREs, we analyzed the promoter DNA sequences within 500 base pairs of the transcription start site of all 104 differentially regulated genes in the *mtl1Δ* strain (Supplementary Table S1), to identify potential Msn2/Msn4 binding motifs. Only 19 differentially downregulated genes (i.e. *HSP42, CAR1, HSP104, ALD4, TDH1, SSA4, CTT1, TSL1, HSP78, GPH1, GLK1, PRB1, YML131W, PGM2, YMR196W, TPS2, HSP150, VPS13, YPL260W*) and 6 upregulated genes (i.e. *RPS20, IPI3, SND3, RPL3, PHB2, YBL036C*) contained at least one STRE, indicating that other signaling systems are also involved in regulating the *mtl1Δ* phenotype^17^. In the future, a broader search of all consensus sequences contained in the Yeast Transcription Factor Specificity Compendium database (YeTFaSCo)^57^ is warranted for these differentially regulated genes. Interestingly, inspection of the promoter regions of *WSC1, WSC2, WSC3, MID2*, and *MTL1* revealed that only the *MTL1* promoter contained an STRE, implying that its expression is transcriptionally regulated by TFs Msn2p and Msn4p in response to stress conditions.

### Analysis of metabolome data

The biological effect of a differentially expressed gene can ultimately be manifested in the operation of the biological processes(es) in which it participates in the cell. Thus, a global analysis of cellular metabolites can reveal changes in specific metabolic processes that may be attributed to the absence of a given protein. Also, the biochemical steps being affected targeted within these putative metabolic processes may be identified intuitively. Therefore, to better understand the biological processes that require Mtl1p function(s), a global metabolite analysis was conducted in WT and *mtl1Δ* yeast strains.

**Figure 2:**
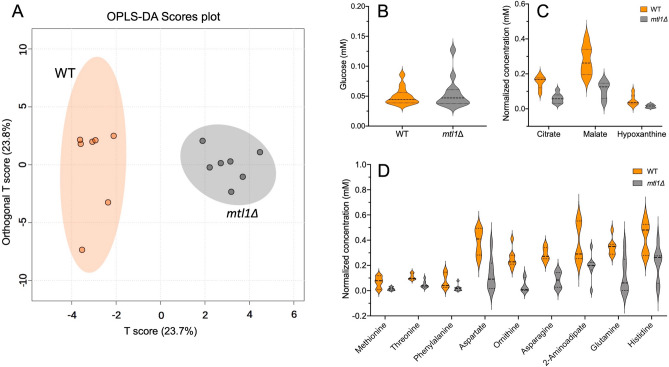
Metabolomics analysis of WT and *mtl1*Δ strains. (**A**) OPLS-DA score plot based on WT and *mtl1*Δ cell metabolomes. (**B**) Violin plots visualize the non-significant distribution of glucose in WT and *mtl1*Δ strains (n = 12). (**C–E**) Violin plots visualize the distribution of significantly altered metabolites in WT and *mtl1*Δ strains (n = 7).

We identified a total of 46 metabolites in this analysis (Table 2). The identification of glucose was conducted separately, and we did not find any differences between WT and *mtl1*Δ strains (Table 2 Row 46, Fig. 2B). The observed fold-change for glucose in *mtl1Δ* was 1.1 (Table 2, Fig. 2B). For a description of the analysis see “Materials and Methods”. The other 45 metabolites extracted from WT and *mtl1*Δ strains were used for metabolomics analysis.

**Table 2:** Difference in metabolite concentrations between WT and *mtl1Δ* strains.

#	ID	Fold change	*p* value
Amino acid metabolism			
1	Alanine	1.01	
2	Asparagine	0.30	0.0006
3	Aspaptate	0.34	0.004
4	Cysteine	0.41	
5	Glutamate	0.75	
6	Glutamine	0.36	0.01
7	Glycine	0.76	
8	Histidine	0.52	0.007
9	Homocysteine	0.57	
10	Homoserine	2.46	
11	Isoleucine	1.06	
12	Leucine	0.77	
13	Methionine	0.21	0.04
14	N-Acetyl-glutamate	0.87	
15	Ornitine	0.16	0.0006
16	Phenylalanine	0.28	0.007
17	Proline	0.48	
18	Serine	0.88	
19	Threonine	0.43	0.007
20	Tryptophan	0.76	
21	Tyrosine	0.72	
22	α-Aminoadipate	0.50	0.007
23	Valine	1.00	
24	5-Oxoprolinate	1.45	
25	5-Oxoproline	0.89	
26	Sarcosine	1.12	

Purine metabolism			
27	Hypoxanthine	0.32	0.0111
28	Orotate	0.34	
29	Uracil	0.82	
30	3-Ureidopropionate	0.98	

Biosynthesis of cysteine			
31	Cystathionine	0.76	

Alpha-hydroxy acid metabolism			
32	Glycolate	0.87	

Saturated fatty acid metabolism			
33	Myristate	0.39	
34	Palmitate	0.77	
35	Stearate	1.24	

Mono-unsaturated fatty acid metabolism			
36	Palmitoleate	0.72	
37	Oleate	0.82	

Poly-unsaturated fatty acid metabolism			
38	Eicosadienoate	1.47	

Organic acid metabolism			
39	Lactate	0.33	

Carboxylic acid metabolism			
40	Citrate	0.40	0.0012
41	Succinate	1.07	
42	Fumarate	1.17	
43	Malate	0.39	0.0006
44	Malonate	0.45	

Vitamine metabolism			
45	Niacinamide	0.69	

Glucose metabolism			
46	Glucose	1.1

The OPLS-DA score plot shows a clear separation between WT and *mtl1*Δ strains (Fig. 2A) with an R2Y of 0.95 and Q2 of 0.78. To further evaluate the model, we performed permutation tests (n = 1000). The empirical *P* values were 0.003 for R2Y and 0.02 for Q2. Thus, a clear distinction between the metabolome of WT and *mtl1*Δ strains was observed (Fig. 2A). Mann–Whitney *U* test analysis detected 12 features significantly decreased in *mtl1Δ* stain compared to WT. (Table 2, Fig. 2C,D). The most significant metabolic changes were related to amino acid metabolism, purine metabolism, and carboxylic acid metabolism.

### Functional enrichment analysis

**Figure 3:**
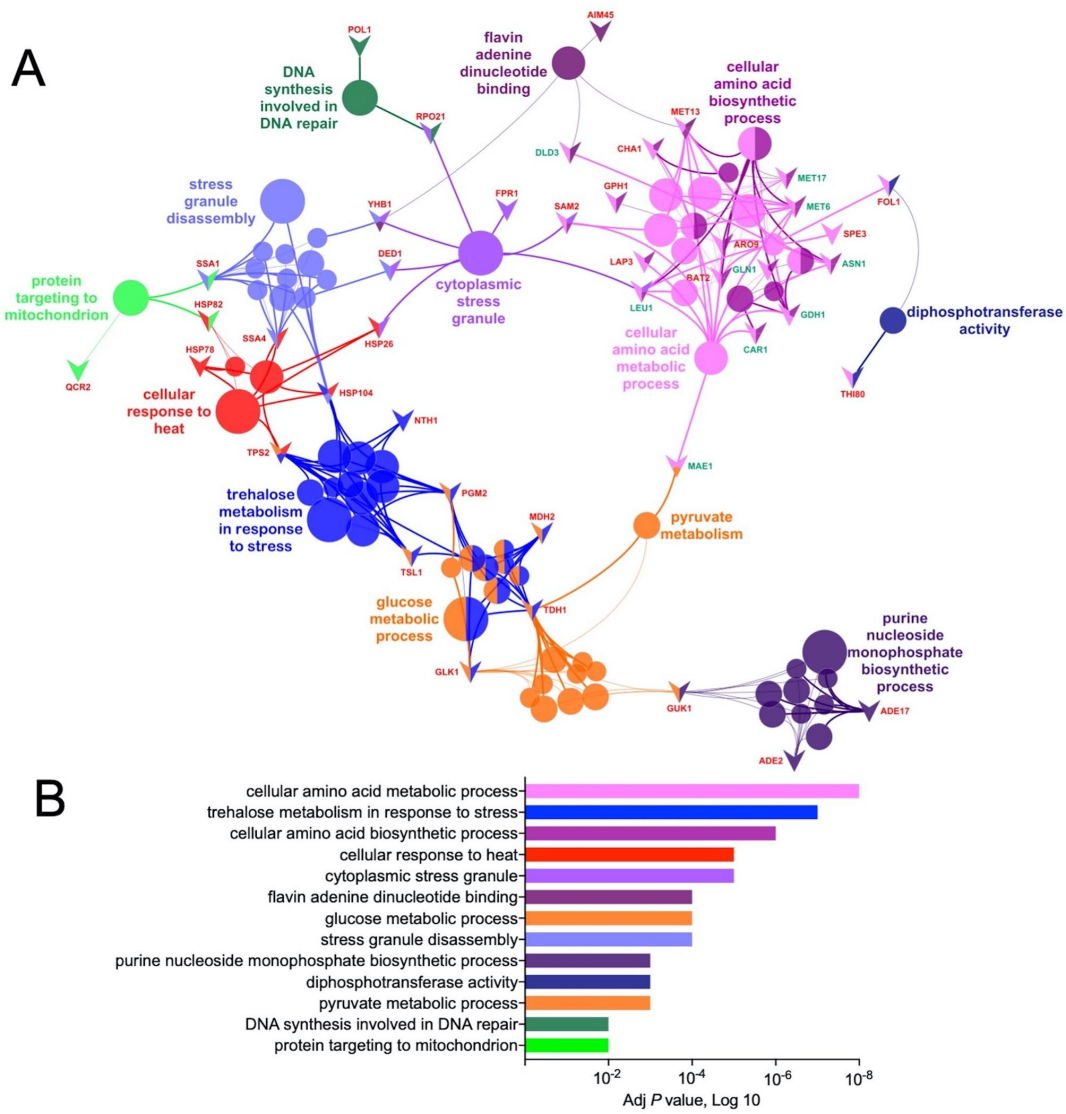
Functional enrichment analysis of the molecular, biological and cellular regulatory mechanisms in the *mtl1Δ* strain. (**A**) Network representations of GO enriched terms in the respective networks in *mtl1*Δ strain of differentially expressed proteins using ClueGO. Enriched terms are represented as circle nodes based on their kappa score (> 0.4) and adjusted *P* values corrected with the Benjamini–Hochberg method. The enrichment significance of the GO terms is reflected by the size of the nodes. Node color represents the class that they belong. Mixed coloring means that the specific node belongs to multiple classes. Associated proteins for each term are presented as triangles. Color of the font indicates upregulation (red) or downregulation (green). The 13 top-ranked categories of GO biological processes are labeled. Sub-network terms for each category are presented in Supplementary Table S2. Visualization has been carried out using ClueGO plugin for Cytoscape 3.7.2. (https://cytoscape.org). (**B**) Top-ranked categories visualized on a bar chart, showing adjusted *P* values (Log10).

The functional enrichment analysis was performed exclusively for groups exhibiting upregulated (Groups 1 and 2) and downregulated (Groups 3 and 4) proteins using ClueGo as described in “Materials and Methods” section. We identified 13 top-ranked categories and their associated proteins including cellular amino acid metabolic and biosynthetic process, trehalose metabolism in response to stress, cellular response to heat, cytoplasmic stress granule, flavin adenine dinucleotide binding, glucose metabolic process, stress granule disassembly, purine nucleoside monophosphate biosynthetic process, diphosphotransferase activity, pyruvate metabolic process, DNA synthesis and protein targeting to mitochondrion (Fig. 3, Supplementary Table S2).

Identified functional enrichments in the cellular response to heat and trehalose metabolism in response to stress in the *mtl1Δ* strain grown at normal conditions and temperature (27 °C) suggest that the deletion of *MTL1* could be associated with the activation of complex regulatory mechanisms related to non-lethal heat stress. Yeast responds to heat stress via rapid synthesis of heat shock proteins (HSP) and trehalose that serves to protect cellular functions by preventing protein aggregation, unfolding aggregated proteins, or targeting denatured proteins for degradation^14^. Thus, we identified significant upregulation of *HSP104, HSP150, HSP26, HSP42, HSP78,* and *HSP82* and enzymes *NTH1, TPS2, TSL1* (Supplementary Table S2, Fig. 3). In addition, trehalose is an effective stabilizer of proteins, suppresses protein aggregation upon denaturation^16^, and acts as an anti-dehydration agent minimizing the effects of dryness^15^. It is recognized that the activity of various trehalose-metabolizing enzymes required the presence of *HSP104* in yeast cells under heat stress^58^. Thus, *HSP104* works synergistically with trehalose to stabilize the yeast proteome in response to heat stress^16^. Moreover, mitochondria have been recognized to play an essential role in heat stress tolerance^59^. In agreement with this assumption, we also observed a functional enrichment in protein targeted to the mitochondrion (Fig. 3). It is recognized that most mitochondrial proteins are synthesized in the cytosol and then translocated to mitochondria to perform their functions. An earlier study showed that heat stress could affect mitochondrial protein translocation efficiency, and therefore, the mitochondrial biosynthesis and functioning are repressed^60^. Our analysis suggests that the *MTL1* deletion did not repress the synthesis of proteins targeted to the mitochondrion since *HSP82*, ubiquinol cytochrome-c reductase, Complex III (QCR2), and *SSA1* were upregulated (Fig. 3).

Functional enrichments in the amino acid metabolic process associated with upregulation in catabolic enzymes *BAT2, ARO9, DUR1,2, MET13* and *CHA1* (Supplementary Table S1 Groups 1—2) and amino acid biosynthetic process concomitant with the downregulation of anabolic enzymes *MET6, MET17, LEU1, GLN1, GDH1, MAE1, ASN1, SAM2, SPE3,* and *CAR1* (Supplementary Table S1 Groups 3–4) suggest that the biosynthesis of amino acids was partially suppressed in the *mtl1Δ* strain. Pairing proteome and metabolome data produced results consistent with this assumption and explained a significant reduction of several metabolites specifically: *CHA1*/threonine, *ARO9*/phenylalanine, *ASN1*/asparagine, *MET6-MET17*-*MET13*/methionine, *GLN1*/glutamine, and *CAR1*/ornithine (Supplementary Table S1, Figs. 1, and 2D). Moreover, identified significant decrease in α-aminoadipate suggests a negative regulation of the anabolic function of the α-aminoadipic acid pathway in yeast, which is used for lysine biosynthesis^61^. A recent study highlights that heat stress in yeast exhibits an anaplerotic character by elevating the expression of a large number of proteins to guarantee constant protein levels for important metabolic processes as a compensative regulatory mechanism against protein aggregation and shutdown of cell growth^14^. In contrast, we observed that the *mtl1Δ* strain displays a cataplerotic character by counterbalancing stress-related detrimental effects by downregulation of anabolic functions.

Previously, it was reported that heat stress could trigger reliance on glycolysis for energy generation and increase the signaling pathway for glucose-regulated gene expression^62^. Accordingly, the glucose metabolic process was significantly enriched by the deletion of *MTL1*. Glyceraldehyde-3-phosphate dehydrogenase (*TDH1*), glucokinase (*GLK1*), phosphoglucomutase (*PGM2*), the regulatory subunit of the trehalose synthase complex (*TSL1*), aldehyde dehydrogenase (*ALD4*) and malate dehydrogenase (*MDH2*) were associated with the glucose metabolic process (Supplementary Table S2). However, we have not found any changes in glucose content in the *mtl1Δ* compared to the WT strain (Table 2, Fig. 2B) presumably because of elevated conversion of glucose to trehalose^63^ by *TPS2* and *TSL1* or pyruvate by *TDH1* (Supplementary Table S2). The fact that pyruvate metabolic process was also significantly enriched in the *mtl1Δ* strain supports our assumption*.* Moreover, it is known that heat stress induces pyruvate accumulation and at the same time, a reduction of pyruvate consumption genes as the first line of cellular defense mechanisms in *S. cerevisiae*^64^. Pyruvate efficiently scavenges heat-induced ROS, which results in a reduction of protein carbonylation, stabilization of the mitochondrial membrane potential (ΔΨm) generated by Complexes I, III and IV during electron transport^64^. Consistent with this study, we observed upregulation of glucokinase (*GLK1*), which converts glucose to glucose-6-phosphate for further glycolytic processing to yield pyruvate as the end product, and downregulation of D-lactate dehydrogenase (*DLD3*), that is coupled to the reduction of pyruvate to lactate. Consequently, a decrease in lactate was identified that could lead to pyruvate accumulation in *mtl1*Δ strain (Table 2). However, the downregulation of mitochondrial malic enzyme (*MAE1*) suggests that oxidative decarboxylation of malate to pyruvate is abolished presumably due to significantly low content of malate (Table 2, Fig. 2C). Elevation of *ALD4* suggests that pyruvate is metabolized by decarboxylation to acetaldehyde via the alternate mitochondrial pyruvate dehydrogenase bypass pathway, and converted to acetate by *ALD4*^65^ which can be further converted to acetyl-CoA. Additionally, it was shown that heat stress depressed the conversion of glucose to glutamate and glutamine, derived from α-ketoglutarate^62^. As a result, both mRNAs and proteins of glutamate dehydrogenase (*GDH1*) and glutamine synthetase (*GLN1*) were downregulated, and correspondently the content of glutamate and glutamine were reduced in the *mtl1Δ* strain (Table 2, Fig. 2D).

**Figure 4:**
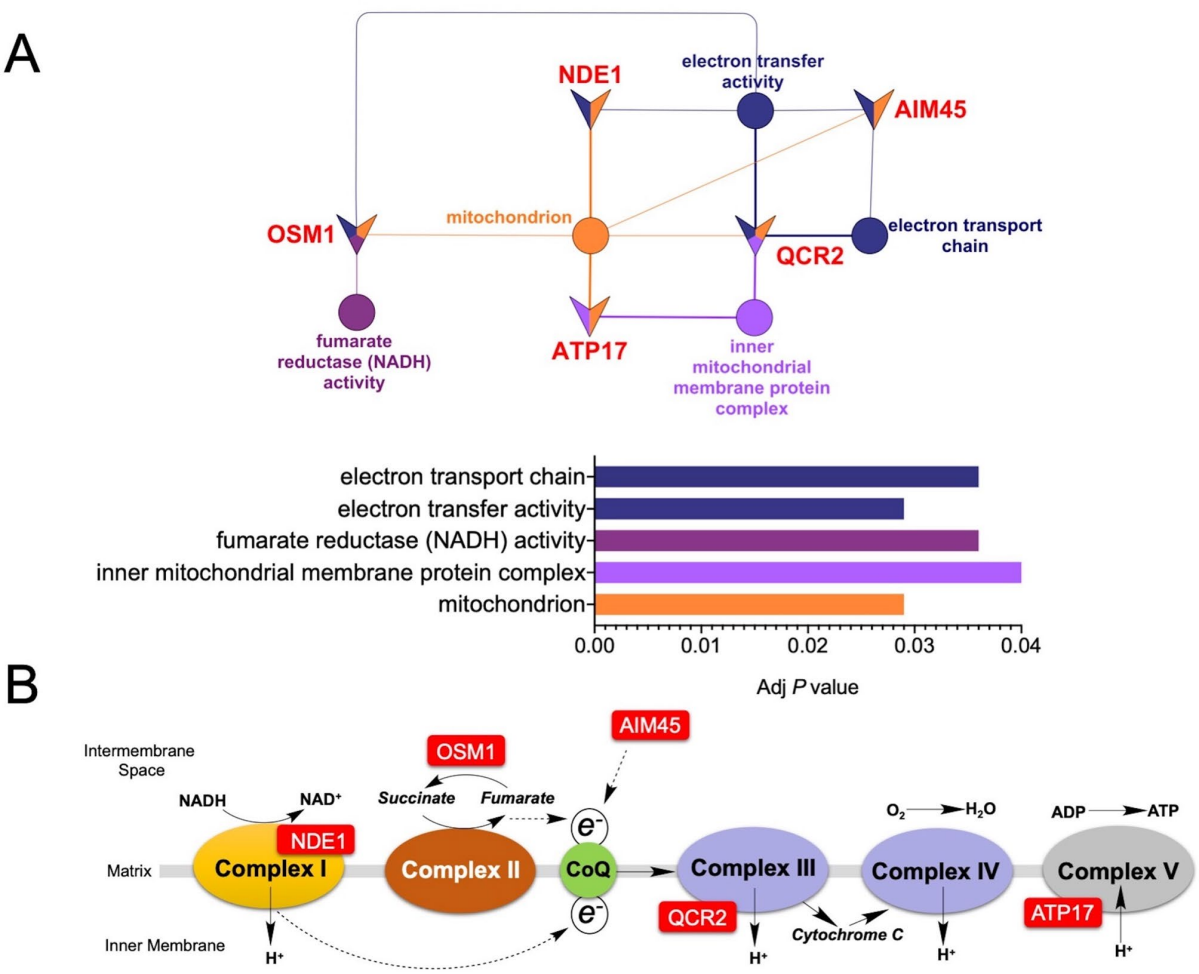
Functional enrichment analysis of the *mtl1Δ* strain respirasome. (**A**) Network representations of GO enriched terms in the respective networks of differentially expressed proteins in the *mtl1Δ* strain using ClueGO. Enriched terms are represented as circle nodes based on their kappa score (> 0.4) and adjusted *P* values corrected with the Benjamini–Hochberg method visualized on a bar chart. Associated proteins for each term are presented as triangles. Node color represents the class that they belong. Mixed coloring means that the specific node belongs to multiple classes. Visualization has been carried out using ClueGO plugin for Cytoscape 3.7.2 (https://cytoscape.org). (**B**) electron transport chain (ETC) depicted associations of identified proteins with ETC complexes. Visualization has been carried out using ChemDraw 15 (PerkinElmer) https://www.perkinelmer.com/category/chemdraw.

An earlier study suggested that in *S. cerevisiae*, heat stress induces oxidative stress through ROS produced mainly by the heat-damaged mitochondrial respirasome and a set of genes associated with the mitochondrial respiratory chain was downregulated^65^. Functional enrichment analysis of the *mtl1Δ* strain respirasome showed a positive correlation of proteins in Supplementary Table S1, Group 2 such as NADH dehydrogenase, Complex I (*NDE1*), Subunit 2 of ubiquinol cytochrome-c reductase (*QCR2*, Complex III) and F1F0 ATP synthase, Complex V (*ATP17*) with mitochondria respirasome (Fig. 4A,B). Moreover, enrichment in flavin adenine dinucleotide binding GO term concomitant with an upregulation of electron transfer flavoprotein *AIM45* that serves as a specific electron acceptor for several dehydrogenases, suggests that *AIM45* can interact with dehydrogenases, including succinate dehydrogenase to convey electrons to ubiquinone in the *mtl1Δ* strain (Fig. 4B). In addition, we identified the upregulation of fumarate oxidoreductase that reduces fumarate to succinate (*OSM1*). The previous study suggested that the *OSM1*/fumarate couple accepts electrons with similar efficiency as cytochrome C^66^. Since we did not find any significant differences in the content of succinate and fumarate (Table 2) in the *mtl1Δ* and WT strains, it suggests that the *mtl1Δ* strain has developed an adaptive response to maintain oxidative phosphorylation at a controlled level to support cell growth. Besides its role in oxidative phosphorylation, *OSM1* is involved in protein targeting to the mitochondrial pathway which is usually activated to prevent protein aggregation, misfolding, or proteolysis triggered by stress^67^.

Furthermore, we identified functional enrichments in the other specific target pathways related to stress-resistance, such as DNA synthesis involved in DNA repair, purine nucleoside monophosphate biosynthetic process, and diphosphotransferase activity (Fig. 3). It is recognized that enzymatic DNA repair pathways become evolved to cope with the heat stress, which can trigger nuclear mutation frequencies and DNA strand breaks^68^. In the *mtl1Δ* strain, we found upregulations of two proteins involved in DNA repair such as the largest subunit B220 of RNAPII (*RPO21*) and DNA polymerase alpha catalytic subunit A (*POL1*) (Supplementary Table S1, Group 2). DNA repair mechanisms involve either tolerating the damage or protecting of the DNA by removing bases, nucleotides, mismatches, homologous recombinations and non-homologous end-joining to ensure overall survival. A nucleotide excision repair mechanism includes transcription-coupled DNA repair when nucleotides are rapidly removed in the transcribed DNA strand^69^. It is known that *RPO21* promotes the intrinsic capacity of RNAPII for transcription bypass of DNA lesions by incorporation or misincorporation of nucleotides across the lesions^70^. *POL1* is a component of the replisome, a multi-component enzymatic machine at the replication fork that mediates DNA replication and specifically modulates both imprecise non-homologous end-joining and more complex chromosomal rearrangements^71^.

In Group 2, we also found upregulation of adenylosuccinate synthetase (*ADE2*) and bifunctional phosphoribosylaminoimidazolecarboxamide formyltransferase/IMP cyclohydrolase (*ADE17*) which along with *GUK1* (Group 1) are associated with purine nucleoside monophosphate biosynthetic process (Fig. 3, and Supplementary Table S2). Purine and pyrimidine nucleotides serve many important functions in cells as precursors to DNA and RNA, energy source, signaling molecules, and cofactor components^72^. De novo purine nucleotide biosynthesis in yeast involves the 10-step production of the purine nucleotide inosine monophosphate (IMP), the common precursor to both AMP and guanosine monophosphate GMP. *ADE2* and *ADE17* are involved in the de novo IMP biosynthetic process while *GUK1* acts in biosynthetic pathways downstream of GMP and AMP (Supplementary Table S2). Since IMP can also be synthesized from hypoxanthine by the salvage pathway, the possibility of incorporating hypoxanthine into DNA and RNA due to heat stress can cause significant defects in purine nucleotide metabolism^73^. Our metabolomics analysis identified a significant reduction in hypoxanthine in the *mtl1*Δ strain presumed to be the result of triggering a complex cellular survival adaptative response to the *MTL1* deletion (Fig. 2C, Table 2). Moreover, we observed a decrease in uracil (Table 2), which, together with hypoxanthine are miscoding and mutagenic in DNA and can interfere with RNA editing and function^73,74^. Identified enrichments of differentially expressed folic acid synthesis protein (*FOL1*) and thiamine pyrophosphokinase (*THI80*) at both protein and RNA levels (Supplementary Table S1, Group 1) associated to the diphosphotransferase activity GO term (Fig. 3) also suggests the triggering of cellular survival adaptive response to the *MTL1* deletion. In particular, *FOL1* is important for the biosynthesis of purines and is required to manage folate deficiency during the heat stress for preventing of massive incorporation of uracil into the DNA^75^. *THI80* produces thiamine pyrophosphate from thiamine—an essential coenzyme for several enzymes in carbohydrate metabolic pathways required for yeast's metabolic adaptation to heat stress and regulation of energy production^59^.

In addition, we observed functional enrichment in cytoplasmic stress granule GO term (Supplementary Table S2, Fig. 3). Stress granules are non-membrane-enclosed RNA granules that dynamically sequester the non-translating messenger ribonucleoproteins, and a large variety of misfolded proteins through protein–protein interactions which vary under different conditions^76–78^. In the *mtl1Δ* strain, we identified upregulations of *HSP26*, known for its role in sequestration of misfolded proteins to stress-induced foci^79,80^, and RNA helicase (*DED1*)—a regulator of 48S translation pre-initiation complex assembly^81^, which directly responds to environmental stress^82^. *DED1* protein rapidly becomes insoluble by forming gel-like condensates that selectively repress translation of housekeeping mRNAs to promote survival under conditions of severe heat stress^82^.

In addition, our functional enrichment analysis predicted associations of five other proteins that were not yet recognized for their associations with cytoplasmic stress granule assembly, including peptidyl-prolyl cis–trans isomerase (*FPR1*), *RPO21,* flavohemoglobin nitric oxide oxidoreductase *(YHB1), LEU1* and *SAM2* (Figs. 1, 3, Supplementary Table S2). However, proteomics analysis of stress granules coupled with super-resolution microscopy in yeast conducted by another group^83^ revealed the occurrence of physical interactions between *DED1* and *YHB1*, *RPO21*, *LEU1*, and *SAM2*, suggesting the possibility that these proteins can be recruited to stress granules. Thus, physical interactions of *YHB1* with *DED1* could explain its important role in oxidative and nitrosative stress responses^84^. Selective degradation of non-functional or unassembled forms of *RPO21* by the 20S proteasome^85^ after completion of RNAPII assembly in the cytoplasm^86^ and interaction of *RPO21* with *DED1* could represent its possible recruitment to stress granules as another quality-control mechanism to prevent *RPO21* accumulation. Moreover, observed downregulation of *SAM2* and *LEU1* followed by a decrease in amino acids methionine and leucine correspondently (Table 2, Fig. 2D) could be sensed by the *mtl1Δ* strain as a stress and as such also triggers *SAM2* and *LEU1* interaction with *DED1* and their recruitment to the stress granules. In addition, functional enrichment analysis predicted the association of *FPR1* with stress granules assembly. *FPR1* is a yeast orthologue of the human FKBP12, which belongs to a family of protein folding chaperones^87^. It was reported that *FPR1* is significantly upregulated when yeast cells are exposed to proteotoxicity-induced stress^88^. In silico modeling showed a possibility of polymerization of FKBP12 with its interacting partner FRB into gel-like structures inside living cells^89^. However, the role of *FPR1* in the process of stress granules formation in yeast is still not completely elucidated.

Functional enrichment in the cytoplasmic stress granule disassembly GO term was concomitant with upregulations of *HSP104* and *SSA1* (Supplementary Table S1, Fig. 3). *HSP104* is the most crucial thermotolerance-related heat shock protein of *Saccharomyces cerevisia*e, which has the ability to rescue denatured proteins through disassembly of high–molecular weight aggregates^90^. *HSP104* is abundant under normal growth conditions but is substantially induced by heat stress^91^. Interestingly, a recent study showed a novel mitochondrial function in preventing protein aggregation in response to heat stress, which could also trigger assembly of protein aggregates on the mitochondrial surface, where *HSP104* facilitates their untangling and transport into the mitochondrial matrix for degradation^92^. *SSA1* is a member of *HSP70* family^90^ which can colocalize with *HSP104* in stress granules and might influence stress granules assembly or disassembly enabling efficient protein homeostasis^80^. Several upregulated proteins including *PHB2, HSP82, SSA4, HSP78, CCT3* act in de novo posttranslational protein folding, while *HSP78* and *HSP82,* can also induce protein refolding. It was also found that *HSP82* is significantly expressed only during heat stress^91^. The accumulation of a significant number of misfolded proteins could have secondary consequences, such as the inhibition of normal protein degradation by the ubiquitin–proteasome system that is controlled in the *mtl1*Δ strain by upregulation of deubiquitinating enzymes *RPT3* and *UBP6*, which spare ubiquitin from proteasomal degradation^93^.

In addition, stress granules are typically observed when cytoplasmic translation is depressed^94^. Accordingly, we detected a significant downregulation of several cytoplasmic ribosomal proteins (Fig. 1, Supplementary Table S1 Group 4), such as *RPL33B, RPL42A, RPS6B* and *RPL3* (large ribosomal subunits), and *RPS1A*, and *RPS20* (small ribosomal subunits), and RSP5 involved in ribosome stability. A recent study suggested that protein degradation and aggregation induced by heat stress can delay the proteomic response^14^, which could explain the weak correlation of the transcriptome and the proteome (Fig. 1, Supplementary Table S1 Group 4). Importantly, downregulation of *RPL33B, RPL42A, RPS6B, RPL3*, *RPS1A*, and *RPS20* coupled with upregulation of the component of the mitochondrial ribosome *MRPL7* (Fig. 1, Supplementary Table S1 Group 1), that suggests a potential molecular switch from cytoplasmic to mitochondrial translation as part of a complex adaptive response to avoid proteotoxic stress that could impact growth of the *mtl1Δ* strain (Supplementary Table S2).

Taken together, an analysis of functional enrichment of the *mtl1Δ* proteome and metabolome suggests that the loss of Mtl1p function can activate complex regulatory mechanisms related to non-lethal heat stress, even under normal culture conditions at 27 °C that disrupts numerous metabolic processes and cellular structures. Based on a preliminary conclusion, *mtl1Δ* strains develop an adaptive response for survival and growth.

### Modeling of TORC1 contribution to the *mtl1Δ* interactome

The *MTL1* function is required to maintain ribosomal gene repression, the general stress response through Rho1p, the inhibition of the TORC1, and activation of the cell wall integrity CWI pathway in response to both glucose starvation and oxidative stress. Since TORC1 functionality was not inhibited in the our *mtl1*Δ strains, it is able to regulate a variety of anabolic and catabolic processes upon sensing diverse nutrient-derived signals including amino acids such as glutamine, leucine, asparagine, arginine and methionine^95–97^. These amino acids, excluding arginine, which was not detected in our samples, were lower in the *mtl1*Δ strains and asparagine and methionine significantly decreased (Table 2, Fig. 2D). Correspondently, anabolic enzymes encoded by *GLN1, ASN1, MET6,* and *MET17* were significantly decreased at both transcriptional and translation levels (Supplementary Table S1, Group 3). Thus, we hypothesize that certain levels of the aforementioned amino acids required to maintain the *mtl1*Δ cellular homeostasis is being provided via an amino acid transporter system^97^, allowing TORC1 to sense their levels and trigger appropriate adaptive responses^98^.

**Figure 5:**
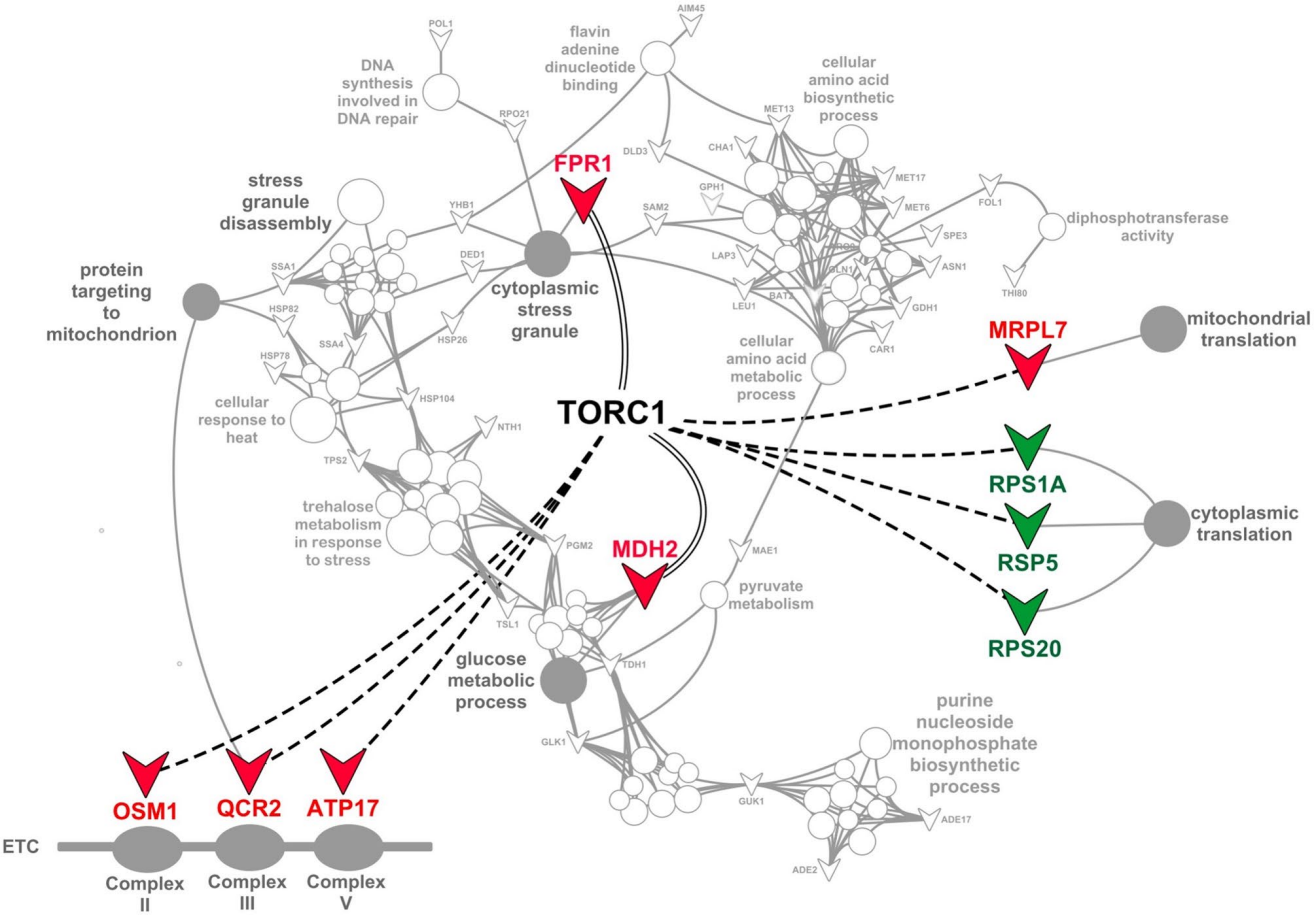
Predicted contribution of TORC1 in the *mtl1Δ* strain interactome. Figure shows the overlay of predicted physical interactions (straight parallel black lines) and genetic interactions (dotted black lines) with GO terms (Fig. 3). Associated upregulated proteins (red) and downregulated proteins (green) are presented as triangles. GO terms concomitant with the predicted TORC1-protein interactions are represented as grey circle nodes. Non-related GO terms and proteins are shown in white. Visualization has been carried out using ClueGO plugin for Cytoscape 3.7.2 (https://cytoscape.org).

Using the GeneMANIA plugin in Cytoscape, we conducted the TORC1 interactome analysis to identify if significantly regulated proteins could interact with TORC1 physically or genetically. Two proteins *FPR1* and *MDH2* potentially interact physically with TORC1 (Fig. 5). *FPR1* is a protein of the FKBP12 family in *S. cerevisiae,* which is a primary target for rapamycin^99^. Addition of rapamycin results in the formation of the FPR1-rapamycin complex required for TORC1 inhibition^100^. Although *FPR1* is recognized to play a crucial role in rapamycin's efficacy, its physiological functions remain unclear. Nevertheless, a previous study suggests that *FPR1* interacts with TORC1 and heat shock proteins Hsp90p and Hsf1p during stress^101^. Consequently, the predicted physical interaction of TORC1 and *FPR1* in our study might explain the observed functional enrichment in the cytoplasmic stress granules pathway components (Fig. 5, Supplementary Table S2). *MDH2* catalyzes the reversible oxidation of malate to oxaloacetate in the citric acid cycle and plays a pivotal role in the malate-aspartate shuttle that operates in the metabolic coordination between cytosol and mitochondria. Since it is known that TORC1 interacts with *MDH2* to protect it from degradation during nutrient stress^69^, the predicted physical interaction of TORC1 with *MDH2* could be the result of adaptive metabolic changes in the *mtl1Δ* strain that resulted in functional enrichment of the glucose metabolic process (Fig. 5). Furthermore, GeneMANIA analysis predicted genetic interaction between the TORC1 and *QCR2, OSM1, ATP17, MRPL7, RSP5, RPS1A,* and *RPS20* encoded proteins (Fig. 5). Gene interaction is a broad term used to describe the joint role of multiple genes on biological processes and phenotypic effects^102^. It is recognized that mammalian TORC1 is a positive regulator of key genes encoding electron transport chain proteins and stimulates oxidative phosphorylation^103^. Given that TORC1 is a highly conserved eukaryotic protein, observed upregulation of *QCR2, OSM1* and *ATP17* and genetic interaction with TORC1 supports our hypothesis of a synergistic association of TORC1 with *QCR2, OSM1* and *ATP17* in shaping the respiratory process in the *mtl1*Δ strain (Fig. 4B, Fig. 5) and protein targeting to the mitochondrial pathway associated with OSM1. Moreover, this analysis predicted a suppressive genetic interaction of TORC1 with *RPS1A, RPS20, RSP5,* and a synergistic genetic interaction with *MRPL7* (Fig. 5). Since TORC1 regulates translation and ribosome biogenesis in *S. cerevisiae*^104^, our data support the contribution of TORC1 to the molecular switch from cytosolic to mitochondrial translation as in the *mtl1*Δ strains. In human cultured cells, TORC1 activity is inversely correlated with mitochondrial protein synthesis and respiration regulating mitochondrial biogenesis^105^.

Taken together, we envisage that a deletion of the *MTL1* gene in *S. cerevisiae* can engage TORC1 signaling concomitant with activation of the cytoplasmic stress granules pathway, enhanced glucose metabolism, oxidative phosphorylation, with protein targeting to the mitochondrial pathway and activation of the molecular switch from cytoplasmic to mitochondrial translation.

In summary, a deficiency of the Mtl1 protein in the *mtl1Δ* strain triggers what we describe as a non-lethal heat stress-like response in yeast cells rendering them more sensitive than the WT to oxidative stress caused by H_2_O_2_, as previously described by others^12,31^. Mtl1p is a key mechanosensory protein for recognizing environmental stress by a mechanism that requires its functional and structural integrity to signal the suitability of temperature, pH, carbon source and nutrient availability for normal growth^31^. We provide additional evidence supporting that Mtl1p maintains normal cellular homeostasis through a mechanism regulated by TORC1^31,39^. The impairment in Mtl1p function caused by heat denaturation, oxidative stress, or other chemical and environmental threats to cell integrity interrupts this signaling axis and triggers the activation of a general stress response^17^ that we believe to be more similar to the non-lethal heat stress-like response described here for *mtl1Δ* strains. We propose that the responses observed in the *mtl1Δ* mutant mimic a chronic non-lethal heat stress state evidenced by upregulation in the accumulation of heat shock proteins and trehalose synthesis, both of which are factors implicated in the adaptive response to heat stress^106^. Similarly, with the accumulation of cytoplasmic catalase T (22-fold), the enhanced accumulation of proteins that promote cytoplasmic stress granule formation, the increase in enzymes required for amino acid catabolism with a reduction in ribosome biogenesis. The enhanced susceptibility of the *mtl1Δ* mutant to H_2_O_2_ treatment, despite the massive accumulation of catalase T, could be caused by high endogenous levels of ROS^107^. Normally, ROS are mainly produced within the mitochondria^108^. However, proteome and metabolite analysis of the *mtl1Δ* strain showed upregulation in proteins of the mitochondrial respirasome as indicators that this mitochondrial function was not affected by the *MTL1* deletion. This assumption can be tested by direct measurements of mitochondrial physiological activity. Therefore, the enhanced sensitivity to oxidative stress associated with *mtl1Δ* strains^31^ may originate from accumulated ROS generated by other dysregulated metabolic processes. Enhanced catabolism of amino acids and ER stress may represent two potential sources for these ROS.

## Conclusions

We have provided new metabolic data showing that an *mtl1Δ* strain upregulates the accumulation of heat shock proteins and trehalose synthesis, which are known indicators of the adaptive response to non-lethal heat stress that we propose for these strains. Similarly, the accumulation of cytoplasmic catalase T (22-fold), enhanced accumulation of proteins that promote cytoplasmic stress granule formation and enzymes required for amino acid catabolism, combined with a reduction in ribosome biogenesis and increased synthesis of mitochondrial respiratory proteins, indicate a complex cellular stress response characterized by elements of heat stress and nutrient deprivation. Without having found evidence of mitochondrial malfunction, we propose that the downregulation of ribosomal protein synthesis coupled with the upregulation in synthesis of mitochondrial proteins may represents a shift in translation of cytoplasmic proteins to translation of mitochondrial proteins as an adaptive response to Mtl1p deficiency. The combined data supports the new function of active Mtl1p under normal cellular conditions which is to suppress the non-lethal heat stress response presumably via down-regulation of the *MTL1* gene at the transcriptional level followed by triggering an adaptive cellular response to maintain normal metabolic homeostasis.

## Supplementary Information

Supplementary Information.

## Data Availability

Metabolomics data are available at the NIH Common Fund's National Metabolomics Data Repository (NMDR) website, the Metabolomics Workbench^109^ (Project ID PR001034, https://doi.org/10.21228/M81X28). RNAseq data are available at Sequence Read Archive (SRA) repository, with the accession number PRJNA686365 (https://www.ncbi.nlm.nih.gov/sra/?term=PRJNA686365). Proteomics data are available at the Proteomics Identifications Database (PRIDE). Currently, it is only accessible for authors and reviewers (to access the data please go to https://www.ebi.ac.uk/pride/archive/login, and use these credentials to login User: reviewer_pxd023963@ebi.ac.uk, Password: oPr4sZae, then look for the accession number PXD023963). The data will be publicly accessible after the paper is accepted for publication.
