# Supplementary material for: Toward the discovery of biological functions associated with the mechanosensor Mtl1p of Saccharomyces cerevisiae via integrative multi-OMICs analysis
Source: Sci Rep. 2021 Apr 1;11:7411. doi: 10.1038/s41598-021-86671-8 (PMC8016984; doi:10.1038/s41598-021-86671-8)
Supplement: Supplementary file 1 — Supplementary Information. [file 41598_2021_86671_MOESM1_ESM.docx]

Toward the discovery of biological functions associated with the mechanosensor Mtl1p of *Saccharomyces cerevisiae* via integrative multi-OMICs analysis.

Nelson Martínez–Matías^1^, Nataliya Chorna^2^, Sahily González–Crespo^1^, Lilliam Villanueva^1^, Ingrid Montes–Rodríguez^3^, Loyda M. Melendez-Aponte^4^, Abiel Roche–Lima^5^, Kelvin Carrasquillo–Carrión^5^, Ednalise Santiago-Cartagena^6^, Brian C. Rymond^7^, Mohan Babu^8^, Igor Stagljar^9,10^, and José R. Rodríguez–Medina^1*^

1. Department of Biochemistry, University of Puerto Rico, Medical Sciences Campus, San Juan, PR 00936-5067
2. Puerto Rico-INBRE Metabolomics Core Facility, Department of Biochemistry, University of Puerto Rico, Medical Sciences Campus, San Juan, PR 00936-5067

3. University of Puerto Rico, Comprehensive Cancer Center, Puerto Rico Medical

Center, Rio Piedras, PR 0093X

4. Department of Microbiology and Medical Zoology, University of Puerto Rico,

Medical Sciences Campus, San Juan, PR 00936-5067

5. Integrated Informatics Services Core (IIS-RCMI), University of Puerto Rico, Medical

Sciences Campus, San Juan, PR 00936-5067

6. School of Pharmacy, University of Puerto Rico, Medical Sciences Campus, San Juan,

PR 00936-5067

7. Department of Biology, University of Kentucky, Lexington, KY 40506

8. Department of Biochemistry, University of Regina, Regina, Saskatchewan, S4S 0A2,

Canada

9. Donnelly Centre, Department of Biochemistry, Department of Molecular Genetics,

University of Toronto, Ontario M5S 3E1, Canada

10. Mediterranean Institute for Life Sciences, Split, Croatia

*Correspondence should be addressed: José R. Rodríguez–Medina, Ph.D. and Nataliya Chorna, Ph.D.; Department of Biochemistry, University of Puerto Rico, Medical Sciences Campus, San Juan, PR 00936-5067; E-mail: [jose.rodriguez123@upr.edu](mailto:jose.rodriguez123@upr.edu) ; Office Telephone 787-758-2525 ext. 2299 and [nataliya.chorna@upr.edu](mailto:nataliya.chorna@upr.edu); Office Telephone 787-758-2525 ext. 1640.


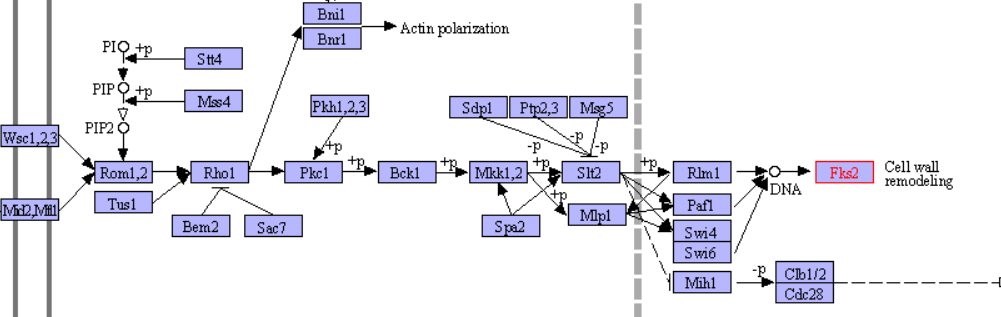


**Fks1,2**

Supplementary Figure S1. Transcriptional upregulation of Fks1p, involved in the cell wall remodeling pathway, requires activation of the Pkc1p activator Rho1p. An increase in accumulation of Fks1 and Fks2 proteins was

observed in the *mtl1Δ* strains without increasing mRNA accumulation suggesting that post-transcriptional levels

of regulation (taken from KEGG Pathway https://www.genome.jp/keg bin/show_pathway?ko04011+K00706,

visited 08-28-2020).

Supplementary Table S1. Proteomic and RNAseq analyses performed on *mtl1Δ* vs wild-type yeast strains.

N=5 for each strain in Proteomic experiments ; N=3 for each strain in RNAseq experiments*.*

|  |  |  |  |  |  |  |
| --- | --- | --- | --- | --- | --- | --- |
|  |  | ***Proteomic Analysis*** | | ***RNAseq Analysis*** | |  |
| **Uniprot_Accession** | **Uniprot_Gene_Symbol** | **FC** | **P-value** | **FC** | **P-value** |  |
| **Group 1** |  |  |  |  |  |  |
| P14743 | NMT1 | 8.41 | 3.65E-03 | 2.48 | 2.15E-02 | N-myristoyl transferase, catalyzes the cotranslational, covalent attachment of myristic acid to the N-terminal glycine residue of several proteins involved in cellular growth and signal transduction Source:SGD;Acc:S000004185 |
| P15454 | GUK1 | 7.59 | 5.19E-03 | 2.73 | 9.88E-04 | Guanylate kinase, converts GMP to GDP; required for growth and mannose outer chain elongation of cell wall N-linked glycoproteins Source:SGD;Acc:S000002862 |
| P20081 | FPR1 | 6.88 | 8.93E-03 | 2.49 | 4.47E-02 | Peptidyl-prolyl cis-trans isomerase (PPIase), binds to the drugs FK506 and rapamycin; also binds to the nonhistone chromatin binding protein Hmo1p and may regulate its assembly or function Source:SGD;Acc:S000005079 |
| P20840 | SAG1 | 19.51 | 9.19E-03 | 3.91 | 5.10E-05 | Alpha-agglutinin of alpha-cells, binds to Aga1p during agglutination, N-terminal half is homologous to the immunoglobulin superfamily and contains binding site for a-agglutinin, C-terminal half is highly glycosylated and contains GPI anchor Source:SGD;Acc:S000003764 |
| P21242 | PRE10 | 4.57 | 5.27E-03 | 2.58 | 1.12E-02 | Alpha 7 subunit of the 20S proteasome Source:SGD;Acc:S000005889 |
| P21375 | OSM1 | 9.99 | 1.09E-02 | 2.66 | 2.04E-02 | Fumarate reductase, catalyzes the reduction of fumarate to succinate, required for the reoxidation of intracellular NADH under anaerobic conditions; mutations cause osmotic sensitivity Source:SGD;Acc:S000003812 |
| P25379 | CHA1 | 91.94 | 5.46E-05 | 2.43 | 4.18E-02 | Catabolic L-serine (L-threonine) deaminase, catalyzes the degradation of both L-serine and L-threonine; required to use serine or threonine as the sole nitrogen source, transcriptionally induced by serine and threonine Source:SGD;Acc:S000000569 |
| P30605 | ITR1 | 13.06 | 3.17E-02 | 2.63 | 4.65E-02 | Myo-inositol transporter with strong similarity to the minor myo-inositol transporter Itr2p, member of the sugar transporter superfamily; expression is repressed by inositol and choline via Opi1p and derepressed via Ino2p and Ino4p Source:SGD;Acc:S000002905 |
| P32804 | ZRT1 | 89.58 | 5.93E-06 | 5.00 | 1.04E-04 | High-affinity zinc transporter of the plasma membrane, responsible for the majority of zinc uptake; transcription is induced under low-zinc conditions by the Zap1p transcription factor Source:SGD;Acc:S000003224 |
| P33204 | ARC19 | 3.23 | 2.11E-02 | 3.21 | 5.77E-03 | Subunit of the ARP2/3 complex, which is required for the motility and integrity of cortical actin patches Source:SGD;Acc:S000001496 |
| P33298 | RPT3 | 6.58 | 2.22E-02 | 2.56 | 2.14E-02 | One of six ATPases of the 19S regulatory particle of the 26S proteasome involved in the degradation of ubiquitinated substrates; substrate of N-acetyltransferase B Source:SGD;Acc:S000002802 |
| P33331 | NTF2 | 6.94 | 3.30E-02 | 2.87 | 4.14E-04 | Nuclear envelope protein, interacts with GDP-bound Gsp1p and with proteins of the nuclear pore to transport Gsp1p into the nucleus where it is an essential player in nucleocytoplasmic transport Source:SGD;Acc:S000000811 |
| P33753 | TRM2 | 8.64 | 6.63E-03 | 2.61 | 1.10E-02 | tRNA methyltransferase, 5-methylates the uridine residue at position 54 of tRNAs and may also have a role in tRNA stabilization or maturation; endo-exonuclease with a role in DNA repair Source:SGD;Acc:S000001764 |
| P33754 | SEC66 | 11.00 | 1.27E-02 | 2.73 | 5.30E-03 | Non-essential subunit of Sec63 complex (Sec63p, Sec62p, Sec66p and Sec72p); with Sec61 complex, Kar2p/BiP and Lhs1p forms a channel competent for SRP-dependent and post-translational SRP-independent protein targeting and import into the ER Source:SGD;Acc:S000000375 |
| P35202 | THI80 | 11.50 | 2.37E-04 | 2.66 | 2.37E-02 | Thiamine pyrophosphokinase, phosphorylates thiamine to produce the coenzyme thiamine pyrophosphate (thiamine diphosphate) Source:SGD;Acc:S000005669 |
| P36519 | MRPL7 | 13.40 | 3.34E-03 | 2.89 | 1.59E-03 | Mitochondrial ribosomal protein of the large subunit; MRPL7 produces both YmL5 and YmL7, which are two different modified forms of the same protein Source:SGD;Acc:S000002645 |
| P39077 | CCT3 | 4.99 | 2.88E-02 | 2.52 | 1.68E-02 | Subunit of the cytosolic chaperonin Cct ring complex, related to Tcp1p, required for the assembly of actin and tubulins in vivo Source:SGD;Acc:S000003551 |
| P39676 | YHB1 | 9.31 | 2.06E-03 | 3.00 | 1.23E-04 | Nitric oxide oxidoreductase, flavohemoglobin involved in nitric oxide detoxification; plays a role in the oxidative and nitrosative stress responses Source:SGD;Acc:S000003466 |
| P40016 | RPN3 | 5.27 | 6.02E-03 | 2.40 | 4.70E-02 | Essential, non-ATPase regulatory subunit of the 26S proteasome lid, similar to the p58 subunit of the human 26S proteasome; temperature-sensitive alleles cause metaphase arrest, suggesting a role for the proteasome in cell cycle control Source:SGD;Acc:S000000823 |
| P38821 | APE4 | 8.78 | 1.72E-03 | 2.57 | 1.22E-02 | Cytoplasmic aspartyl aminopeptidase that may also function in the vacuole; CVT pathway cargo protein; cleaves unblocked N-terminal acidic amino acids from peptide substrates; forms a 12-subunit homo-oligomer; M18 metalloprotease family Source:SGD;Acc:S000001155 |
| P43593 | UBP6 | 8.75 | 2.92E-02 | 2.43 | 3.75E-02 | Ubiquitin-specific protease situated in the base subcomplex of the 26S proteasome, releases free ubiquitin from branched polyubiquitin chains; works in opposition to Hul5p polyubiquitin elongation activity; mutant has aneuploidy tolerance Source:SGD;Acc:S000001906 |
| P50085 | PHB2 | 8.90 | 5.91E-03 | 2.50 | 1.63E-02 | Subunit of the prohibitin complex (Phb1p-Phb2p), a 1.2 MDa ring-shaped inner mitochondrial membrane chaperone that stabilizes newly synthesized proteins; determinant of replicative life span; involved in mitochondrial segregation Source:SGD;Acc:S000003463 |
| P50108 | MNN10 | 10.74 | 1.26E-02 | 2.56 | 6.79E-03 | Subunit of a Golgi mannosyltransferase complex also containing Anp1p, Mnn9p, Mnn11p, and Hoc1p that mediates elongation of the polysaccharide mannan backbone; membrane protein of the mannosyltransferase family Source:SGD;Acc:S000002653 |
| P53848 | FOL1 | 10.12 | 3.50E-02 | 2.63 | 1.79E-02 | Multifunctional enzyme of the folic acid biosynthesis pathway, has dihydropteroate synthetase, dihydro-6-hydroxymethylpterin pyrophosphokinase, and dihydroneopterin aldolase activities Source:SGD;Acc:S000005200 |
| P53877 | IPI3 | 12.24 | 8.82E-04 | 2.80 | 8.33E-04 | Essential component of the Rix1 complex (Rix1p, Ipi1p, Ipi3p) that is required for processing of ITS2 sequences from 35S pre-rRNA; highly conserved and contains WD40 motifs; Rix1 complex associates with Mdn1p in pre-60S ribosomal particles Source:SGD;Acc:S000005126 |
| Q01532 | LAP3 | 7.50 | 1.31E-03 | 2.47 | 1.84E-02 | Cysteine aminopeptidase with homocysteine-thiolactonase activity; protects cells against homocysteine toxicity; has bleomycin hydrolase activity in vitro; transcription is regulated by galactose via Gal4p; orthologous to human BLMH Source:SGD;Acc:S000005183 |
| Q03305 | RMT2 | 5.20 | 2.09E-02 | 2.54 | 2.76E-02 | Arginine N5 methyltransferase; methylates ribosomal protein Rpl12 (L12) on Arg67 Source:SGD;Acc:S000002873 |
| Q03774 | TRM82 | 4.34 | 1.40E-02 | 2.80 | 1.89E-03 | Catalytic subunit of a tRNA methyltransferase complex; Trm8p and Trm82p comprise an enzyme that catalyzes a methyl-transfer from S-adenosyl-l-methionine to the N(7) atom of guanine at position 46 in tRNA; Trm8 lacks catalytic activity if not bound t /.../2p Source:SGD;Acc:S000002572 |
| Q04225 | RRB1 | 3.47 | 3.40E-02 | 2.54 | 1.16E-02 | Essential nuclear protein involved in early steps of ribosome biogenesis; physically interacts with the ribosomal protein Rpl3p Source:SGD;Acc:S000004738 |
| Q06405 | ATP17 | 15.66 | 2.72E-04 | 2.95 | 1.20E-02 | Subunit f of the F0 sector of mitochondrial F1F0 ATP synthase, which is a large, evolutionarily conserved enzyme complex required for ATP synthesis Source:SGD;Acc:S000002785 |
| Q12117 | MRH1 | 5.46 | 1.14E-02 | 2.69 | 6.82E-03 | Protein that localizes primarily to the plasma membrane, also found at the nuclear envelope; the authentic, non-tagged protein is detected in mitochondria in a phosphorylated state; has similarity to Hsp30p and Yro2p Source:SGD;Acc:S000002440 |
| Q12155 | YLR050C | 12.58 | 1.59E-02 | 3.06 | 5.42E-03 | Putative protein of unknown function; green fluorescent protein (GFP)-fusion protein localizes to the endoplasmic reticulum; YLR050C is not an essential gene Source:SGD;Acc:S000004040 |
| Q12480 | AIM45 | 6.92 | 2.23E-02 | 2.56 | 9.50E-03 | Putative ortholog of mammalian electron transfer flavoprotein complex subunit ETF-alpha; interacts with frataxin, Yfh1p; null mutant displays elevated frequency of mitochondrial genome loss; may have a role in oxidative stress response Source:SGD;Acc:S000006208 |
| **Group 2** |  |  |  |  |  |  |
| P38009 | ADE17 | 15.52 | 6.79E-03 | -4.86 | 1.87E-05 | Enzyme of 'de novo' purine biosynthesis containing both 5-aminoimidazole-4-carboxamide ribonucleotide transformylase and inosine monophosphate cyclohydrolase activities, isozyme of Ade16p; ade16 ade17 mutants require adenine and histidine Source:SGD;Acc:S000004727 |
| P21264 | ADE2 | 5.12 | 2.60E-03 | -2.68 | 5.73E-03 | Phosphoribosylaminoimidazole carboxylase, catalyzes a step in the 'de novo' purine nucleotide biosynthetic pathway; red pigment accumulates in mutant cells deprived of adenine Source:SGD;Acc:S000005654 |
| P46367 | ALD4 | 12.24 | 1.40E-02 | -12.24 | 0.00E+00 | Mitochondrial aldehyde dehydrogenase, required for growth on ethanol and conversion of acetaldehyde to acetate; phosphorylated; activity is K+ dependent; utilizes NADP+ or NAD+ equally as coenzymes; expression is glucose repressed Source:SGD;Acc:S000005901 |
| Q01217 | ARG5,6 | 7.51 | 2.03E-02 | -3.07 | 6.27E-05 | Protein that is processed in the mitochondrion to yield acetylglutamate kinase and N-acetyl-gamma-glutamyl-phosphate reductase, which catalyze the 2nd and 3rd steps in arginine biosynthesis; enzymes form a complex with Arg2p Source:SGD;Acc:S000000871 |
| P38840 | ARO9 | 37.98 | 1.22E-02 | -12.72 | 2.84E-06 | Aromatic aminotransferase II, catalyzes the first step of tryptophan, phenylalanine, and tyrosine catabolism Source:SGD;Acc:S000001179 |
| P47176 | BAT2 | 22.62 | 2.11E-06 | -2.45 | 4.83E-02 | Cytosolic branched-chain amino acid (BCAA) aminotransferase, preferentially involved in BCAA catabolism; homolog of murine ECA39; highly expressed during stationary phase and repressed during logarithmic phase Source:SGD;Acc:S000003909 |
| P22137 | CHC1 | 6.06 | 1.52E-03 | -2.96 | 1.50E-04 | Clathrin heavy chain, subunit of the major coat protein involved in intracellular protein transport and endocytosis; two heavy chains form the clathrin triskelion structural component; the light chain (CLC1) is thought to regulate function Source:SGD;Acc:S000003174 |
| P06115 | CTT1 | 22.74 | 1.74E-03 | -7.33 | 1.91E-08 | Cytosolic catalase T, has a role in protection from oxidative damage by hydrogen peroxide Source:SGD;Acc:S000003320 |
| P06634 | DED1 | 3.36 | 4.65E-02 | -2.46 | 3.26E-02 | ATP-dependent DEAD (Asp-Glu-Ala-Asp)-box RNA helicase, required for translation initiation of all yeast mRNAs; mutations in human DEAD-box DBY are a frequent cause of male infertility Source:SGD;Acc:S000005730 |
| P32528 | DUR1,2 | 13.39 | 1.62E-02 | -3.02 | 4.65E-05 | Urea amidolyase, contains both urea carboxylase and allophanate hydrolase activities, degrades urea to CO2 and NH3; expression sensitive to nitrogen catabolite repression and induced by allophanate, an intermediate in allantoin degradation Source:SGD;Acc:S000000412 |
| P17709 | GLK1 | 8.58 | 2.20E-02 | -4.86 | 1.44E-06 | Glucokinase, catalyzes the phosphorylation of glucose at C6 in the first irreversible step of glucose metabolism; one of three glucose phosphorylating enzymes; expression regulated by non-fermentable carbon sources Source:SGD;Acc:S000000545 |
| P06738 | GPH1 | 8.94 | 2.75E-04 | -5.13 | 4.81E-10 | Non-essential glycogen phosphorylase required for the mobilization of glycogen, activity is regulated by cyclic AMP-mediated phosphorylation, expression is regulated by stress-response elements and by the HOG MAP kinase pathway Source:SGD;Acc:S000006364 |
| P31539 | HSP104 | 16.85 | 1.49E-05 | -15.33 | 1.03E-10 | Heat shock protein that cooperates with Ydj1p (Hsp40) and Ssa1p (Hsp70) to refold and reactivate previously denatured, aggregated proteins; responsive to stresses including: heat, ethanol, and sodium arsenite; involved in PSI+ propagation Source:SGD;Acc:S000003949 |
| P32478 | HSP150 | 11.40 | 1.20E-02 | -2.64 | 1.25E-02 | O-mannosylated heat shock protein that is secreted and covalently attached to the cell wall via beta-1,3-glucan and disulfide bridges; required for cell wall stability; induced by heat shock, oxidative stress, and nitrogen limitation Source:SGD;Acc:S000003695 |
| P15992 | HSP26 | 37.82 | 1.19E-04 | -30.64 | 1.73E-14 | Small heat shock protein (sHSP) with chaperone activity; forms hollow, sphere-shaped oligomers that suppress unfolded proteins aggregation; long-lived protein that is preferentially retained in mother cells and forms cytoplasmic foci; oligomer activation requires heat-induced conformational change; also has mRNA binding activity |
| Q12329 | HSP42 | 19.42 | 3.36E-02 | -25.05 | 7.38E-13 | Small heat shock protein (sHSP) with chaperone activity; forms barrel-shaped oligomers that suppress unfolded protein aggregation; involved in cytoskeleton reorganization after heat shock Source:SGD;Acc:S000002578 |
| P33416 | HSP78 | 26.02 | 7.22E-03 | -6.50 | 4.54E-08 | Oligomeric mitochondrial matrix chaperone that cooperates with Ssc1p in mitochondrial thermotolerance after heat shock; able to prevent the aggregation of misfolded proteins as well as resolubilize protein aggregates Source:SGD;Acc:S000002666 |
| P02829 | HSP82 | 13.24 | 1.40E-04 | -3.16 | 3.24E-04 | Hsp90 chaperone required for pheromone signaling and negative regulation of Hsf1p; docks with Tom70p for mitochondrial preprotein delivery; promotes telomerase DNA binding and nucleotide addition; interacts with Cns1p, Cpr6p, Cpr7p, Sti1p Source:SGD;Acc:S000006161 |
| P22133 | MDH2 | 7.62 | 3.94E-04 | -2.47 | 4.68E-02 | Cytoplasmic malate dehydrogenase, one of three isozymes that catalyze interconversion of malate and oxaloacetate; involved in the glyoxylate cycle and gluconeogenesis during growth on two-carbon compounds; interacts with Pck1p and Fbp1 Source:SGD;Acc:S000005486 |
| P53128 | MET13 | 3.68 | 2.18E-02 | -2.66 | 6.97E-03 | Major isozyme of methylenetetrahydrofolate reductase, catalyzes the reduction of 5,10-methylenetetrahydrofolate to 5-methyltetrahydrofolate in the methionine biosynthesis pathway Source:SGD;Acc:S000003093 |
| P40215 | NDE1 | 6.89 | 1.45E-03 | -2.77 | 1.14E-03 | Mitochondrial external NADH dehydrogenase, a type II NAD(P)H:quinone oxidoreductase that catalyzes the oxidation of cytosolic NADH; Nde1p and Nde2p provide cytosolic NADH to the mitochondrial respiratory chain Source:SGD;Acc:S000004753 |
| P32356 | NTH1 | 7.64 | 3.38E-02 | -2.90 | 3.08E-04 | Neutral trehalase, degrades trehalose; required for thermotolerance and may mediate resistance to other cellular stresses; may be phosphorylated by Cdc28p Source:SGD;Acc:S000002408 |
| Q02785 | PDR12 | 19.76 | 9.94E-05 | -3.72 | 7.12E-06 | Plasma membrane ATP-binding cassette (ABC) transporter, weak-acid-inducible multidrug transporter required for weak organic acid resistance; induced by sorbate and benzoate and regulated by War1p; mutants exhibit sorbate hypersensitivity Source:SGD;Acc:S000005979 |
| P37012 | PGM2 | 24.18 | 2.58E-05 | -3.04 | 1.51E-03 | Phosphoglucomutase, catalyzes the conversion from glucose-1-phosphate to glucose-6-phosphate, which is a key step in hexose metabolism; functions as the acceptor for a Glc-phosphotransferase Source:SGD;Acc:S000004711 |
| P13382 | POL1 | 7.48 | 9.61E-03 | -2.48 | 4.19E-02 | Catalytic subunit of the DNA polymerase I alpha-primase complex, required for the initiation of DNA replication during mitotic DNA synthesis and premeiotic DNA synthesis Source:SGD;Acc:S000005046 |
| P09232 | PRB1 | 22.73 | 7.13E-07 | -4.43 | 9.98E-11 | Vacuolar proteinase B (yscB), a serine protease of the subtilisin family; involved in protein degradation in the vacuole and required for full protein degradation during sporulation; activity inhibited by Pbi2p Source:SGD;Acc:S000000786 |
| P07257 | QCR2 | 13.37 | 5.76E-04 | -2.64 | 4.19E-03 | Subunit 2 of the ubiquinol cytochrome-c reductase complex, which is a component of the mitochondrial inner membrane electron transport chain; phosphorylated; transcription is regulated by Hap1p, Hap2p/Hap3p, and heme Source:SGD;Acc:S000006395 |
| P04050 | RPO21 | 4.15 | 3.87E-02 | -2.74 | 4.78E-03 | RNA polymerase II largest subunit B220, part of central core; phosphorylation of C-terminal heptapeptide repeat domain regulates association with transcription and splicing factors; similar to bacterial beta-prime Source:SGD;Acc:S000002299 |
| P38804 | RTC3 | 21.80 | 1.85E-02 | -8.89 | 5.41E-08 | Protein of unknown function involved in RNA metabolism; has structural similarity to SBDS, the human protein mutated in Shwachman-Diamond Syndrome (the yeast SBDS ortholog = SDO1); null mutation suppresses cdc13-1 temperature sensitivity Source:SGD;Acc:S000001129 |
| P48415 | SEC16 | 7.07 | 2.02E-02 | -2.45 | 3.51E-02 | COPII vesicle coat protein required for ER transport vesicle budding; Sec16p is bound to the periphery of ER membranes and may act to stabilize initial COPII complexes; interacts with Sec23p, Sec24p and Sec31p Source:SGD;Acc:S000006006 |
| P10591 | SSA1 | 10.62 | 1.62E-04 | -4.68 | 3.07E-06 | ATPase involved in protein folding and nuclear localization signal (NLS)-directed nuclear transport; member of heat shock protein 70 (HSP70) family; forms a chaperone complex with Ydj1p; localized to the nucleus, cytoplasm, and cell wall; 98% identi /.../th Ssa2p, but subtle differences between the two proteins provide functional specificity with respect to propagation of yeast URE3 prions and vacuolar-mediated degradations of gluconeogenesis enzymes Source:SGD;Acc:S000000004 |
| P22202 | SSA4 | 16.69 | 7.55E-04 | -7.69 | 1.65E-04 | Heat shock protein that is highly induced upon stress; plays a role in SRP-dependent cotranslational protein-membrane targeting and translocation; member of the HSP70 family; cytoplasmic protein that concentrates in nuclei upon starvation Source:SGD;Acc:S000000905 |
| P00360 | TDH1 | 63.38 | 7.64E-06 | -9.75 | 6.48E-12 | Glyceraldehyde-3-phosphate dehydrogenase, isozyme 1, involved in glycolysis and gluconeogenesis; tetramer that catalyzes the reaction of glyceraldehyde-3-phosphate to 1,3 bis-phosphoglycerate; detected in the cytoplasm and cell wall Source:SGD;Acc:S000003588 |
| Q03280 | TOM1 | 7.20 | 2.57E-02 | -2.51 | 2.05E-02 | E3 ubiquitin ligase of the hect-domain class; has a role in mRNA export from the nucleus and may regulate transcriptional coactivators; involved in degradation of excess histones Source:SGD;Acc:S000002865 |
| P31688 | TPS2 | 8.09 | 7.98E-03 | -2.80 | 9.68E-04 | Phosphatase subunit of the trehalose-6-phosphate synthase/phosphatase complex, which synthesizes the storage carbohydrate trehalose; expression is induced by stress conditions and repressed by the Ras-cAMP pathway Source:SGD;Acc:S000002481 |
| P38427 | TSL1 | 14.90 | 7.34E-06 | -7.06 | 4.45E-07 | Large subunit of trehalose 6-phosphate synthase (Tps1p)/phosphatase (Tps2p) complex, which converts uridine-5'-diphosphoglucose and glucose 6-phosphate to trehalose, similar to Tps3p and may share function; mutant has aneuploidy tolerance Source:SGD;Acc:S000004566 |
| Q07878 | VPS13 | 12.64 | 4.80E-04 | -2.52 | 2.12E-02 | Protein of unknown function; heterooligomeric or homooligomeric complex; peripherally associated with membranes; homologous to human COH1; involved in sporulation, vacuolar protein sorting and protein-Golgi retention Source:SGD;Acc:S000003963 |
| P40483 | YIL108W | 7.93 | 1.07E-02 | -2.52 | 2.30E-02 | Putative metalloprotease Source:SGD;Acc:S000001370 |
| Q03102 | YML131W, | 9.88 | 5.81E-03 | -3.13 | 1.09E-03 | Putative protein of unknown function with similarity to medium chain dehydrogenase/reductases; expression induced by stresses including osmotic shock, DNA damaging agents, and other chemicals; GFP-fusion protein localizes to the cytoplasm Source:SGD;Acc:S000004600 |
| Q04336 | YMR196W | 9.40 | 2.14E-03 | -2.99 | 2.72E-03 | Putative protein of unknown function; green fluorescent protein (GFP)-fusion protein localizes to the cytoplasm; YMR196W is not an essential gene Source:SGD;Acc:S000004809 |
| P38631 | FKS1,2 | 7.07 | 1.33E-03 | -2.52 | 2.46E-02 | 1,3-beta-glucan synthase component FKS1 (EC 2.4.1.34) (1,3-beta-D-glucan-UDP glucosyltransferase) (Calcineurin dependent protein 1) (Calcofluor white hypersensitivity protein 53) (Echinocandin target gene protein 1) (FK506 sensitivity protein 1) (Glucan synthase of cerevisiae protein 1) (Papulacandin B resistance protein 1) |
| Q08977 | YPL260W | 4.46 | 4.18E-03 | -2.44 | 2.91E-02 | Putative substrate of cAMP-dependent protein kinase (PKA); green fluorescent protein (GFP)-fusion protein localizes to the cytoplasm and nucleus; YPL260W is not an essential gene Source:SGD;Acc:S000006181 |
| **Group 3** |  |  |  |  |  |  |
| P00812 | CAR1 | -22.78 | 2.97E-04 | -22.84 | 9.50E-11 | Arginase, catabolizes arginine to ornithine and urea; expression responds to both induction by arginine and nitrogen catabolite repression; disruption decreases production of carcinogen ethyl carbamate during wine fermentation and also enhances freeze tolerance |
| P05694 | MET6 | -8.65 | 6.20E-05 | -2.78 | 3.28E-03 | Cobalamin-independent methionine synthase, involved in methionine biosynthesis and regeneration; requires a minimum of two glutamates on the methyltetrahydrofolate substrate, similar to bacterial metE homologs Source:SGD;Acc:S000000893 |
| P06106 | MET17 | -16.40 | 8.55E-05 | -3.09 | 3.38E-05 | Methionine and cysteine synthase (O-acetyl homoserine-O-acetyl serine sulfhydrylase), required for sulfur amino acid synthesis Source:SGD;Acc:S000004294 |
| P07262 | GDH1 | -7.84 | 1.20E-02 | -3.29 | 1.56E-04 | NADP(+)-dependent glutamate dehydrogenase, synthesizes glutamate from ammonia and alpha-ketoglutarate; rate of alpha-ketoglutarate utilization differs from Gdh3p; expression regulated by nitrogen and carbon sources Source:SGD;Acc:S000005902 |
| P07264 | LEU1 | -14.20 | 1.99E-03 | -2.97 | 5.08E-04 | Isopropylmalate isomerase, catalyzes the second step in the leucine biosynthesis pathway Source:SGD;Acc:S000002977 |
| P28834 | IDH1 | -4.92 | 1.63E-02 | -4.43 | 6.67E-06 | Subunit of mitochondrial NAD(+)-dependent isocitrate dehydrogenase, which catalyzes the oxidation of isocitrate to alpha-ketoglutarate in the TCA cycle Source:SGD;Acc:S000004982 |
| P32288 | GLN1 | -8.37 | 8.47E-05 | -4.39 | 5.47E-08 | Glutamine synthetase (GS), synthesizes glutamine from glutamate and ammonia; with Glt1p, forms the secondary pathway for glutamate biosynthesis from ammonia; expression regulated by nitrogen source and by amino acid limitation Source:SGD;Acc:S000006239 |
| P36013 | MAE1 | -3.50 | 4.25E-02 | -3.80 | 7.19E-06 | Mitochondrial malic enzyme, catalyzes the oxidative decarboxylation of malate to pyruvate, which is a key intermediate in sugar metabolism and a precursor for synthesis of several amino acids Source:SGD;Acc:S000001512 |
| P39940 | RSP5 | -21.50 | 6.06E-06 | -2.72 | 2.34E-03 | E3 ubiquitin ligase of the NEDD4 family; involved in regulating many cellular processes including MVB sorting, heat shock response, transcription, endocytosis, and ribosome stability; human homolog is involved in Liddle syndrome; mutant tolerates an /.../dy; ubiquitylates Sec23p Source:SGD;Acc:S000000927 |
| P39976 | DLD3 | -9.88 | 1.14E-02 | -2.78 | 1.01E-03 | D-lactate dehydrogenase, part of the retrograde regulon which consists of genes whose expression is stimulated by damage to mitochondria and reduced in cells grown with glutamate as the sole nitrogen source, located in the cytoplasm Source:SGD;Acc:S000000797 |
| P49089 | ASN1 | -11.62 | 5.27E-03 | -3.56 | 4.21E-06 | Asparagine synthetase, isozyme of Asn2p; catalyzes the synthesis of L-asparagine from L-aspartate in the asparagine biosynthetic pathway Source:SGD;Acc:S000006349 |
| Q06689 | YLR413W | -7.61 | 9.23E-05 | -2.64 | 1.73E-02 | Putative protein of unknown function; YLR413W is not an essential gene Source:SGD;Acc:S000004405 |
| **Group 4** |  |  |  |  |  |  |
| P08466 | NUC1 | -7.67 | 3.24E-02 | 2.86 | 2.03E-03 | Major mitochondrial nuclease, has RNAse and DNA endo- and exonucleolytic activities; has roles in mitochondrial recombination, apoptosis and maintenance of polyploidy Source:SGD;Acc:S000003744 |
| P0CX27 | RPL42A | -7.21 | 3.75E-02 | 3.67 | 1.65E-02 | Protein component of the large (60S) ribosomal subunit, identical to Rpl42Bp and has similarity to rat L44 ribosomal protein Source:SGD;Acc:S000005106 |
| P0CX38 | RPS6B | -6.82 | 1.65E-02 | 2.56 | 2.76E-02 | Protein component of the small (40S) ribosomal subunit; identical to Rps6Ap and has similarity to rat S6 ribosomal protein Source:SGD;Acc:S000000385 |
| P14126 | RPL3 | -8.23 | 6.55E-04 | 2.59 | 3.57E-02 | Protein component of the large (60S) ribosomal subunit, has similarity to E. coli L3 and rat L3 ribosomal proteins; involved in the replication and maintenance of killer double stranded RNA virus Source:SGD;Acc:S000005589 |
| P18851 | STE4 | -9.57 | 4.51E-02 | 2.47 | 2.00E-02 | G protein beta subunit, forms a dimer with Ste18p to activate the mating signaling pathway, forms a heterotrimer with Gpa1p and Ste18p to dampen signaling; may recruit Rho1p to the polarized growth site during mating; contains WD40 repeats Source:SGD;Acc:S000005738 |
| P19358 | SAM2 | -5.56 | 1.73E-02 | 2.80 | 4.46E-03 | S-adenosylmethionine synthetase, catalyzes transfer of the adenosyl group of ATP to the sulfur atom of methionine; one of two differentially regulated isozymes (Sam1p and Sam2p) Source:SGD;Acc:S000002910 |
| P20107 | ZRC1 | -6.60 | 3.99E-02 | 2.41 | 4.29E-02 | Vacuolar membrane zinc transporter, transports zinc from the cytosol into the vacuole for storage; also has a role in resistance to zinc shock resulting from a sudden influx of zinc into the cytoplasm Source:SGD;Acc:S000004856 |
| P22141 | PRE1 | -7.30 | 1.25E-02 | 2.95 | 1.61E-03 | Beta 4 subunit of the 20S proteasome; localizes to the nucleus throughout the cell cycle Source:SGD;Acc:S000000814 |
| P33442 | RPS1A | -9.86 | 9.33E-03 | 2.60 | 1.59E-02 | Ribosomal protein 10 (rp10) of the small (40S) subunit; nearly identical to Rps1Bp and has similarity to rat S3a ribosomal protein Source:SGD;Acc:S000004433 |
| P38197 | YBL036C | -6.62 | 2.86E-03 | 2.38 | 4.95E-02 | Putative non-specific single-domain racemase based on structural similarity; binds pyridoxal 5'-phosphate; expression of GFP-fusion protein induced in response to the DNA-damaging agent MMS Source:SGD;Acc:S000000132 |
| P38264 | PHO88 | -2.65 | 3.58E-02 | 2.71 | 5.58E-03 | Probable membrane protein, involved in phosphate transport; pho88 pho86 double null mutant exhibits enhanced synthesis of repressible acid phosphatase at high inorganic phosphate concentrations Source:SGD;Acc:S000000310 |
| P38701 | RPS20 | -4.34 | 2.69E-02 | 2.82 | 5.88E-03 | Protein component of the small (40S) ribosomal subunit; overproduction suppresses mutations affecting RNA polymerase III-dependent transcription; has similarity to E. coli S10 and rat S20 ribosomal proteins Source:SGD;Acc:S000001007 |
| P41056 | RPL33B | -5.11 | 2.06E-02 | 2.64 | 1.49E-02 | Ribosomal protein L37 of the large (60S) ribosomal subunit, nearly identical to Rpl33Ap and has similarity to rat L35a; rpl33b null mutant exhibits normal growth while rpl33a rpl33b double null mutant is inviable Source:SGD;Acc:S000005760 |
| P53177 | TYW3 | -8.86 | 4.67E-03 | 2.55 | 4.92E-02 | tRNA methyltransferase required for synthesis of wybutosine, a modified guanosine found at the 3'-position adjacent to the anticodon of phenylalanine tRNA which supports reading frame maintenance by stabilizing codon-anticodon interactions Source:SGD;Acc:S000003018 |
| P53969 | SAM50 | -4.55 | 1.79E-02 | 2.52 | 3.16E-02 | Essential component of the Sorting and Assembly Machinery (SAM or TOB complex) of the mitochondrial outer membrane, which binds precursors of beta-barrel proteins and facilitates their outer membrane insertion; homologous to bacterial Omp85 Source:SGD;Acc:S000004971 |
| Q05905 | HRI1 | -6.02 | 3.97E-02 | 2.62 | 1.22E-02 | Protein of unknown function that interacts with Sec72p and Hrr25p Source:SGD;Acc:S000004292 |
| Q12074 | SPE3 | -5.14 | 1.00E-02 | 2.74 | 3.01E-03 | Spermidine synthase, involved in biosynthesis of spermidine and also in biosynthesis of pantothenic acid; spermidine is required for growth of wild-type cells Source:SGD;Acc:S000006273 |

Supplementary Table S2. Functional enrichment of differentially regulated proteins in *mtl1Δ* strains with their Gene Ontology terms using ClueGo. GO terms highlighted.

| **GOID** | **GOTerm** | **Term PValue** | **Term PValue Corrected with Benjamini-Hochberg** | **Associated Proteins Found** |
| --- | --- | --- | --- | --- |
| **GO:0008652** | **cellular amino acid biosynthetic process*** | **6.10E-07** | **5.20E-06** | **ARO9, ASN1, BAT2, GDH1, GLN1, LEU1, MET13, MET17, MET6** |
| GO:0009084 | glutamine family amino acid biosynthetic process | 1.60E-02 | 1.90E-02 | GDH1, GLN1 |
| GO:0016053 | organic acid biosynthetic process | 2.00E-08 | 3.50E-07 | ARO9, ASN1, BAT2, DLD3, FOL1, GDH1, GLN1, LEU1, MET13, MET17, MET6, SPE3 |
| GO:0030170 | pyridoxal phosphate binding | 3.50E-04 | 8.40E-04 | ARO9, CHA1, GPH1, MET17 |
| GO:0000097 | sulfur amino acid biosynthetic process | 1.60E-05 | 5.90E-05 | ARO9, BAT2, MET13, MET17, MET6 |
| GO:0009067 | aspartate family amino acid biosynthetic process | 3.80E-06 | 2.00E-05 | ARO9, ASN1, BAT2, MET13, MET17, MET6 |
| GO:0009098 | leucine biosynthetic process | 1.60E-03 | 3.00E-03 | BAT2, LEU1 |
| GO:0009086 | methionine biosynthetic process | 1.10E-05 | 4.90E-05 | ARO9, BAT2, MET13, MET17, MET6 |
| **GO:0006520** | **cellular amino acid metabolic process** | **2.90E-09** | **9.80E-08** | **ARO9, ASN1, BAT2, CAR1, CHA1, GDH1,GLN1, LAP3, LEU1, MAE1, MET13, MET17, MET6, SAM2** |
| GO:0009069 | serine family amino acid metabolic process | 4.00E-02 | 4.20E-02 | CHA1, MET17 |
| GO:0000096 | sulfur amino acid metabolic process | 6.20E-08 | 7.00E-07 | ARO9, BAT2, LAP3, MET13, MET17, MET6, SAM2 |
| GO:0006551 | leucine metabolic process | 3.10E-03 | 4.90E-03 | BAT2, LEU1 |
| GO:0006555 | methionine metabolic process | 6.70E-07 | 5.10E-06 | ARO9, BAT2, MET13, MET17, MET6, SAM2 |
| GO:0006541 | glutamine metabolic process | 8.00E-04 | 1.70E-03 | ASN1, GDH1, GLN1 |
| **GO:0000731** | **DNA synthesis involved in DNA repair** | **1.10E-02** | **1.40E-02** | **POL1, RPO21** |
| **GO:0006626** | **protein targeting to mitochondrion** | **1.60E-02** | **1.90E-02** | **HSP82, QCR2, SSA1** |
| **GO:0016778** | **diphosphotransferase activity** | **2.10E-03** | **3.50E-03** | **FOL1, THI80** |
| **GO:0050660** | **flavin adenine dinucleotide binding** | **1.80E-04** | **4.80E-04** | **AIM45, DLD3, MET13, YHB1** |
| **GO:0010494** | **cytoplasmic stress granule** | **1.40E-05** | **5.90E-05** | **DED1, FPR1, HSP26, LEU1, RPO21, SAM2, YHB1** |
| **GO:0035617** | **stress granule disassembly** | **3.50E-04** | **8.20E-04** | **HSP104, SSA1** |
| GO:0051084 | 'de novo' posttranslational protein folding | 6.10E-04 | 1.30E-03 | HSP104, SSA1, SSA4 |
| GO:0051085 | chaperone cofactor-dependent protein refolding | 6.10E-04 | 1.30E-03 | HSP104, SSA1, SSA4 |
| GO:0032988 | ribonucleoprotein complex disassembly | 5.10E-05 | 1.60E-04 | DED1, HSP104, SSA1 |
| GO:0072599 | establishment of protein localization to endoplasmic reticulum | 4.60E-02 | 4.60E-02 | SSA1, SSA4 |
| GO:0045047 | protein targeting to ER | 4.40E-02 | 4.50E-02 | SSA1, SSA4 |
| GO:0006613 | cotranslational protein targeting to membrane | 1.50E-02 | 1.70E-02 | SSA1, SSA4 |
| GO:0006986 | response to unfolded protein | 4.70E-02 | 4.70E-02 | SSA1, SSA4 |
| GO:0006616 | SRP-dependent cotranslational protein targeting to membrane, translocation | 7.50E-03 | 1.00E-02 | SSA1, SSA4 |
| GO:0034620 | cellular response to unfolded protein | 3.30E-02 | 3.50E-02 | SSA1, SSA4 |
| GO:0035967 | cellular response to topologically incorrect protein | 1.20E-02 | 1.50E-02 | SSA1, SSA4, YHB1 |
| **GO:0070413** | **trehalose metabolism in response to stress** | **8.70E-06** | **4.20E-05** | **HSP104, TPS2, TSL1** |
| GO:0005946 | alpha,alpha-trehalose-phosphate synthase complex (UDP-forming) | 5.80E-04 | 1.30E-03 | TPS2, TSL1 |
| GO:0009311 | oligosaccharide metabolic process | 2.80E-06 | 1.60E-05 | HSP104, NTH1, PGM2, TPS2, TSL1 |
| GO:0005984 | disaccharide metabolic process | 1.60E-06 | 1.10E-05 | HSP104, NTH1, PGM2, TPS2, TSL1 |
| GO:0009312 | oligosaccharide biosynthetic process | 3.60E-05 | 1.20E-04 | PGM2, TPS2, TSL1 |
| GO:0005991 | trehalose metabolic process | 1.70E-08 | 3.90E-07 | HSP104, NTH1, PGM2, TPS2, TSL1 |
| GO:0046351 | disaccharide biosynthetic process | 3.60E-05 | 1.20E-04 | PGM2, TPS2, TSL1 |
| GO:0005992 | trehalose biosynthetic process | 2.40E-05 | 8.60E-05 | PGM2, TPS2, TSL1 |
| GO:0019203 | carbohydrate phosphatase activity | 3.70E-03 | 5.60E-03 | TPS2, TSL1 |
| GO:0004805 | trehalose-phosphatase activity | 5.80E-04 | 1.30E-03 | TPS2, TSL1 |
| **GO:0006006** | **glucose metabolic process** | **1.80E-04** | **4.80E-04** | **GLK1, MDH2, PGM2, TDH1** |
| GO:0016051 | carbohydrate biosynthetic process | 2.20E-04 | 5.70E-04 | MDH2, PGM2, TDH1, TPS2, TSL1 |
| GO:0046364 | monosaccharide biosynthetic process | 1.00E-02 | 1.30E-02 | MDH2, TDH1 |
| GO:0019319 | hexose biosynthetic process | 8.40E-03 | 1.10E-02 | MDH2, TDH1 |
| GO:0006094 | gluconeogenesis | 8.40E-03 | 1.10E-02 | MDH2, TDH1 |
| GO:0005996 | monosaccharide metabolic process | 1.70E-03 | 3.00E-03 | GLK1, MDH2, PGM2, TDH1 |
| GO:0019318 | hexose metabolic process | 9.40E-04 | 1.90E-03 | GLK1, MDH2, PGM2, TDH1 |
| GO:0051156 | glucose 6-phosphate metabolic process | 1.10E-02 | 1.40E-02 | GLK1, PGM2 |
| GO:0009132 | nucleoside diphosphate metabolic process | 2.40E-03 | 4.00E-03 | GLK1, GUK1, TDH1 |
| GO:0046939 | nucleotide phosphorylation | 2.40E-02 | 2.60E-02 | GLK1, TDH1 |
| GO:0006165 | nucleoside diphosphate phosphorylation | 2.40E-02 | 2.60E-02 | GLK1, TDH1 |
| GO:0009135 | purine nucleoside diphosphate metabolic process | 1.70E-03 | 3.00E-03 | GLK1, GUK1, TDH1 |
| GO:0009185 | ribonucleoside diphosphate metabolic process | 1.70E-03 | 3.00E-03 | GLK1, GUK1, TDH1 |
| GO:0006757 | ATP generation from ADP | 2.00E-02 | 2.20E-02 | GLK1, TDH1 |
| GO:0009179 | purine ribonucleoside diphosphate metabolic process | 1.70E-03 | 3.00E-03 | GLK1, GUK1, TDH1 |
| GO:0046031 | ADP metabolic process | 2.20E-02 | 2.50E-02 | GLK1, TDH1 |
| GO:0006096 | glycolytic process | 2.00E-02 | 2.20E-02 | GLK1, TDH1 |
| **GO:0006090** | **pyruvate metabolic process** | **3.60E-03** | **5.50E-03** | **GLK1, MAE1, TDH1** |
| **GO:0034605** | **cellular response to heat** | **1.40E-05** | **5.70E-05** | **HSP104, HSP26, HSP78, HSP82, SSA4, TPS2** |
| GO:0009408 | response to heat | 5.30E-05 | 1.60E-04 | HSP104, HSP26, HSP78, HSP82, SSA4, TPS2 |
| GO:0050821 | protein stabilization | 8.40E-03 | 1.10E-02 | HSP78, HSP82 |
| **GO:0009127** | **purine nucleoside monophosphate biosynthetic process** | **9.00E-04** | **1.90E-03** | **ADE17, ADE2, GUK1** |
| GO:0006189 | 'de novo' IMP biosynthetic process | 2.10E-03 | 3.50E-03 | ADE17, ADE2 |
| GO:0009124 | nucleoside monophosphate biosynthetic process | 5.30E-03 | 7.30E-03 | ADE17, ADE2, GUK1 |
| GO:0009126 | purine nucleoside monophosphate metabolic process | 1.10E-03 | 2.20E-03 | ADE17, ADE2, GUK1 |
| GO:0009161 | ribonucleoside monophosphate metabolic process | 5.00E-03 | 7.00E-03 | ADE17, ADE2, GUK1 |
| GO:0009156 | ribonucleoside monophosphate biosynthetic process | 4.40E-03 | 6.30E-03 | ADE17, ADE2, GUK1 |
| GO:0009167 | purine ribonucleoside monophosphate metabolic process | 1.10E-03 | 2.20E-03 | ADE17, ADE2, GUK1 |
| GO:0046040 | IMP metabolic process | 4.40E-03 | 6.50E-03 | ADE17, ADE2 |
| GO:0009168 | purine ribonucleoside monophosphate biosynthetic process | 9.00E-04 | 1.90E-03 | ADE17, ADE2, GUK1 |

* The top-ranked categories of GO biological processes are in bold font and highlighted by grey color. Red- upregulated proteins, Green- downregulated proteins.

Supplementary Table S3. TMT Experiment Design

| **TMT Labels** | | | |
| --- | --- | --- | --- |
| **Tag** | **Reporter Ion** | **Sample Name** | **Sample Group** |
| TMT-126 | 126.127726 | WT 1 | Control Group |
| TMT-127N | 127.124761 | WT 2 |  |
| TMT-127C | 127.131081 | WT 3 |  |
| TMT-128N | 128.128116 | WT 4 |  |
| TMT-128C | 128.134436 | WT 5 |  |
| TMT-129N | 129.131471 | Mtl 1 | Experimental Group |
| TMT-129C | 129.137790 | Mtl 2 |  |
| TMT-130N | 130.134825 | Mtl 3 |  |
| TMT-130C | 130.141145 | Mtl 4 |  |
| TMT-131 | 131.144499 | Mtl 5 |  |
